# Experimental infection of bumblebees with honeybee-associated viruses: no direct fitness costs but potential future threats to novel wild bee hosts

**DOI:** 10.1098/rsos.200480

**Published:** 2020-07-08

**Authors:** Anja Tehel, Tabea Streicher, Simon Tragust, Robert J. Paxton

**Affiliations:** 1 General Zoology, Institute for Biology, Martin Luther University Halle-Wittenberg, Hoher Weg 8, 06120 Halle (Saale), Germany; 2 German Centre for Integrative Biodiversity Research (iDiv) Halle-Jena-Leipzig, Deutscher Platz 5e, 04103 Leipzig, Germany

**Keywords:** *Apis mellifera*, *Bombus terrestris*, deformed wing virus, black queen cell virus, multi-host pathogen, virulence

## Abstract

Pathogen spillover represents an important cause of biodiversity decline. For wild bee species such as bumblebees, many of which are in decline, correlational data point towards viral spillover from managed honeybees as a potential cause. Yet, impacts of these viruses on wild bees are rarely evaluated. Here, in a series of highly controlled laboratory infection assays with well-characterized viral inocula, we show that three viral types isolated from honeybees (deformed wing virus genotype A, deformed wing virus genotype B and black queen cell virus) readily replicate within hosts of the bumblebee *Bombus terrestris*. Impacts of these honeybee-derived viruses - either injected or fed - on the mortality of *B. terrestris* workers were, however, negligible and probably dependent on host condition. Our results highlight the potential threat of viral spillover from honeybees to novel wild bee species, though they also underscore the importance of additional studies on this and other wild bee species under field-realistic conditions to evaluate whether pathogen spillover has a negative impact on wild bee individuals and population fitness.

## 1. Introduction

A wealth of evidence points to massive biodiversity loss in the Anthropocene, resulting in range declines, local extirpations and species extinctions [1]. Though generally considered mobile and numerous, mounting evidence demonstrates that many insect species and communities are also in decline [2–4], with potential consequences for the functioning of terrestrial ecosystems [5]. Bees are a particular focus of concern because of their importance in pollination [6], with strong support for range decline and species loss in temperate regions of the world [7–9]. Causes of bee decline, as for the fate of other insects, are thought to revolve around the fragmentation, degradation and loss of habitat, intensification of land use (including pesticides) and climate change as well as, potentially, pathogens [10–12].

Pathogen spillover is an important cause of biodiversity decline as well as a risk to human health [13]. Recent examples of pathogen spillover causing population decline include European whitenose fungus killing North American bats [14], Asiatic chitrid fungus decimating European populations of the amphibian *Salamandra salamandra* [15] and Ebola virus that spills over from wild mammal reservoir hosts into humans to cause life-threatening disease [16]. Yet, pathogen spillover may have variable, and sometimes benign, consequences for novel hosts. For example, the exotic *Nosema ceranae* microsporidian of the Asiatic honeybee *Apis cerana* is nowadays an emerging infectious disease (EID) of *Apis mellifera* [17] throughout much of the world. It spills over into wild bee species, where it has been reported to reduce the lifespan of the Australian stingless bee *Tetragonula hockingsi* [18], though causes little apparent harm to the Eurasian *Bombus terrestris* [19,20] and European mason bee *Osmia bicornis* [21]. In these cases, the pathogen might be merely vectored through novel bee hosts [22] rather than cause them harm.

There is mounting correlational evidence that the Western honeybee *A. mellifera*, the world's most important commercial pollinator, is a source of pathogens that spill over into wild bee species [19,23–28], in which those pathogens may cause population decline. While black queen cell virus (BQCV) is the most prevalent virus in honeybees [24,26,29,30], temperate regions of the world have seen elevated honeybee colony losses [31], probably caused by the exotic invasive ectoparasitic mite *Varroa destructor* and deformed wing virus (DWV), which the mite transmits [32–34]. DWV is an EID which has become panzootic in honeybee populations to which *V. destructor* has been introduced, i.e. worldwide excluding Australia [35].

Bumblebees (*Bombus* spp.) are widespread wild bee species in northern temperate regions [36], yet many are decreasing in abundance or distribution, with parasites being a potential cause of their decline [37,38]. BQCV is the most prevalent virus in bumblebees [24,26,29,30], it exhibits broad tissue tropism in the American *Bombus huntii* [39,40], and its prevalence in *Bombus* spp. covaries with that in *Apis*. Though *Bombus* spp. are not known to host *V. destructor*, spillover of DWV from honeybees to bumblebees has been inferred from the tight relationship between DWV prevalence in populations of *A. mellifera* and *Bombus* spp. and higher prevalence in the former [19], with pathogen transmission presumably occurring through shared use of flowers [28,41]. DWV is a highly virulent pathogen of *A. mellifera* comprising two main genotypes: the original DWV genotype A (DWV-A) and the more virulent DWV genotype B (DWV-B) [42], both of which have been inferred to spill over from honeybees to bumblebees [19]. A leading hypothesis is that *V. destructor* parasitism of honeybees, by elevating DWV prevalence and intensity of infection (pathogen load) in honeybees, may help drive pathogen spillover from honeybees to bumblebees [27]. We note, though, that most data suggesting viral spillover from honeybees to bumblebees are correlational; directionality has rarely been demonstrated and wild bees may also be a source of infection for honeybees [24].

Two studies have to date evaluated the virulence of DWV to bumblebees. Firstly, Fürst *et al.* [19] found that a mixed DWV-A/DWV-B inoculum fed to caged *B. terrestris* workers led to a significant increase in mortality over 20 days. It is not known whether observed mortality was due to DWV-A, DWV-B, enhanced virulence due to co-infection or an A–B recombinant. Though DWV-A and DWV-B are widespread, have high prevalence in British and US honeybees and often co-occur in the same host [42,43], A–B recombinants were rarely detected in US honeybees [43], suggesting they may be infrequent. However, A–B recombinants have been shown to exhibit elevated virulence in honeybees [44] and may have composed the inoculum of Fürst *et al.* [19]. In the second study, Graystock *et al.* [45] injected DWV derived from *B. terrestris* fat bodies into conspecific, caged workers and revealed a 50% increase in mortality. In this second study [45], DWV was isolated from *B. terrestris* hosts, to which it had potentially adapted, thus not reflecting a spillover scenario from honeybees to bumblebees. In both studies [19,45], viral titre in bumblebees following experimental inoculation was not quantified, making it unclear how well DWV replicated in *B. terrestris* and whether it *per se*, as opposed to a potentially pre-existing pathogen in experimental bees or inoculum, induced elevated mortality.

To clarify the potential impact of honeybee-associated viruses on bumblebees, we experimentally inoculated *B. terrestris* workers with either BQCV, DWV-A or DWV-B derived from honeybees and thereafter quantified host mortality and viral titre. Inoculation of bumblebees was done by injection, so as to determine the capacity of the virus to replicate in a novel host, as well as by feeding, representing the more likely natural route of infection in the field [41]. These experiments were carried out under *ad libitum* food conditions. However, fitness costs when responding to an immune challenge may be dependent on host nutritional state, and have been shown for bumblebees when diet was restricted [46]. We, therefore, complemented our investigation with an experiment under starvation conditions.

## 2. Material and methods

### 2.1. Source of bees

Commercial *B. terrestris* colonies (Koppert B.V., Berkel en Rodenrijs, The Netherlands) were kept in an incubator at 30°C and 50% relative humidity with *ad libitum* 50% (w/v) sucrose solution. Every 2–3 days, they were fed with fresh-frozen honeybee pollen pellets (Imkerei Schachtner, Schardenberg, Austria) that had been freshly defrosted. Pollen was UV-irradiated before use to destroy pathogens. Honeybees for experiments and for generating viral inocula were taken from our local apiary (University of Halle, Germany), originally purchased as the subspecies *Apis mellifera carnica*, as is typical for beekeeping in the region. To check that bumblebees (12 source colonies: labelled B1–B12) and honeybees (2 source colonies, labelled 5.1 and G) as well as the fresh-frozen pollen pellets were devoid of viral pathogens, we tested them by real-time quantitative PCR (qPCR) for seven common honeybee viral targets and three Microsporidia (electronic supplementary material). Bumblebee and honeybee colonies were largely free of virus (electronic supplementary material, table S1), pollen was devoid of virus and Microsporidia were not detected.

### 2.2. Propagation of viral inocula

To propagate DWV-A and DWV-B for experimental inocula, we used the inocula from Tehel *et al.* [47]. Our BQCV inoculum was prepared by propagating the BQCV inoculum of Doublet *et al*. [48]. Viral propagation in honeybee pupae and absolute quantification of virus followed precisely methods in Tehel *et al.* [47]. We always generated the correct virus inoculum from the original inoculum, which was devoid of other viruses (electronic supplementary material, figure S1).

Inocula containing only DWV-A, only DWV-B or only BQCV at known concentrations were aliquoted and stored at –80°C for use in experiments, as was the control inoculum devoid of virus. For each virus, a single inoculum derived from one preparation was used for all experiments with *Bombus* and *Apis*. Ultradeep next-generation sequencing (NGS) on an Illumina platform confirmed the identity of our DWV-A and DWV-B inocula (see [47] for consensus sequences and the pipeline used to assemble them from NGS data as well as BioProject ID PRJNA515220 for the original NGS source files).

### 2.3. Experimental inoculation

#### 2.3.1. Honeybees: injected with inoculum, satiated

We initially ensured that viral inocula were viable by injecting them into honeybee workers.

Freshly eclosed workers were cooled to 4°C and then injected laterally between the second and third tergite with 10^7^ viral genome equivalents (or, as control, virus-free inoculum), a quantity sufficient to ensure 100% infection of adults [42], using a Hamilton syringe (hypodermic needle outer diameter: 0.235 mm). To avoid cross-contamination, syringes were cleaned after each use, and different syringes were used for each inoculum (DWV-A, DWV-B, BQCV) and for the control inoculum devoid of virus. The 249 individually injected honeybees were randomly assigned to injection treatments, held in groups of 20–22 in autoclaved metal cages (10 × 10 × 6 cm) independent of their source colony but with bees of the same treatment per individual cage in an incubator (30°C), fed *ad libitum* with 50% (weight/volume) sucrose solution and monitored daily till death, as in McMahon *et al.* [42]. At 10 days post-inoculation (d.p.i.), one bee per cage was removed to quantify viral titre.

#### 2.3.2. Bumblebees: general handling

Viral inocula were tested in freshly emerged *B. terrestris* workers as follows. Firstly, we marked all workers in our 12 *B. terrestris* colonies. Colonies were checked daily and unmarked, newly emerged workers were transferred to autoclaved metal cages (10 × 10 × 6 cm), fed *ad libitum* with 50% (w/v) sucrose solution and held in an incubator at 30°C. On the next day (i.e. 24–48 h after eclosion), workers were inoculated with virus (or control solution), either by injection or orally by feeding, and then kept in groups of 5–10 of the same treatment per cage. In an experiment, the number of bees per cage was constant (± one bee) for every treatment within any 1 day of infection. This procedure was repeated across 25 days to allow for sufficient replication per experiment.

##### 2.3.2.1. Bumblebees: fed inoculum, satiated

Inoculation of *B. terrestris* workers by feeding was designed to test the likely route of viral spillover from honeybees at flowers in the field. Freshly emerged (24–48 h after eclosion) bumblebee workers were individually fed with 10^9^ viral genome equivalents or the equivalent control solution devoid of virus (electronic supplementary material), a quantity inducing an acute infection [48,49]. Then bees were transferred to a new, autoclaved metal cage in small groups (5–10 bees per cage, grouped according to treatment). In total, 512 bees from five source colonies were evenly distributed between all four treatments (DWV-A, DWV-B, BQCV, control; 128 bumblebees per treatment) and were randomly assigned to cages independent of source colony. They were monitored daily for mortality. One bee per cage was removed at 18–25 d.p.i. to quantify viral titre. In a preliminary trial following the identical protocol as described above, we quantified viral titres at 10 and 20 d.p.i., but found no significant difference among them or with bees tested at 18–25 d.p.i. (electronic supplementary material, figure S2).

##### 2.3.2.2. Bumblebees: injected with inoculum, satiated

Inoculation by injection was designed to test whether *B. terrestris* is a competent host for each virus. To inject workers, they were cooled on ice till immobile. Viral inoculation then followed that for honeybees; *B. terrestris* workers were then transferred to autoclaved metal cages in small groups (five to seven bees per cage). Bees were randomly assigned to cages independent of their four source colonies but grouped according to treatment per cage, resulting in *n* = 404 bees that were recorded daily for mortality (*ca* 100 bees per treatment: DWV-A, DWV-B, BQCV, control). One bee per cage was removed at 10 d.p.i. to quantify viral titre.

##### 2.3.2.3. Bumblebees: injected with inoculum, starved

As *B. terrestris* workers did not exhibit elevated mortality over controls following viral inoculation under benign laboratory conditions with *ad libitum* food (see Results), we ran an additional experiment in which we removed their food to determine whether viral inocula induced mortality under non-benign, starvation conditions. Bees from three colonies were collected over a 14 day period as they eclosed, held in autoclaved metal cages and individually injected as described above. To control statistically for effects of age, bees of approximately the same age were held in the same cage. All bees were injected on the same day. At 13 d.p.i., after the virus had time to replicate, bees were individually transferred to a plastic cup covered with netting, devoid of sucrose solution but with a small cotton wool ball soaked in water, held at 30°C and checked every hour for mortality (electronic supplementary material, figure S3).

At death, bee size was estimated because size might determine the ability to survive under starvation [50] (electronic supplementary material, figure S6). Viral titre was quantified in a subset of bees collected at 13 d.p.i. In total, 326 *B. terrestris* were inoculated by injection in this experiment, of which 194 survived till 13 d.p.i. and, therefore, entered the starvation part of the experiment.

#### 2.3.3. Viral titres

To quantify viral titres in adult worker bees arising from inoculation experiments, we crushed one whole honeybee or one bumblebee abdomen in 500 µl of 0.5 M PPB (pH 8.0) using a plastic pestle, of which 100 µl were used for RNA isolation. Absolute quantification of viral titre followed methods used for viral inocula described in Tehel *et al.* [47] (electronic supplementary material), including all positive and negative controls.

### 2.4. Statistics

All analyses were performed in R v. 3.5.1 (R Core Team). We used generalized linear models (GLMs) with a quasi-Poisson error distribution to test for the effect of treatment or experiment on viral titre.

Survivorship of experimentally inoculated bees was analysed using the Cox proportional hazards models with the R package *coxme* [51,52]. ‘Cage’ was used as a random factor in all analyses and ‘round of infection’ as a random factor for *B. terrestris* experiments in which an experiment was initiated across multiple days. To assess the significance of predictors, statistical models including all predictors were compared with null (intercept only) or reduced models (for those with multiple predictors) using likelihood ratio (LR) tests. Pairwise comparisons between factor levels of a significant predictor were performed using pairwise *post hoc* tests, adjusting the family-wise error rate according to the method of Bonferroni (package *multcomp*, [53]). For the experiments with bumblebees under satiated conditions (inoculated by injection and by feeding), survival models retained ‘cage’ and date or ‘round of infection’ (for *B. terrestris* experiments in which an experiment was initiated across multiple days) as random factors and treatment as a fixed factor. For the *Bombus* experiment under starvation conditions, ‘cage’ was again retained as a random factor, and treatment together with bee age and bee size entered as fixed factors. The median survival was calculated using the Survfit function in *survival*. In all survival analyses, bees that died within 1 day (24 h) post-inoculation were eliminated from subsequent analyses as death was probably a consequence of physical damage by injection *per se* rather than the inoculum.

## 3. Results

### 3.1. Honeybees: injected with inoculum, satiated

All viral inocula, BQCV, DWV-A and DWV-B, resulted in rapid honeybee mortality (electronic supplementary material, figure S4a); which was significantly faster than control (Cox proportional hazard: BQCV, Exp. (*β*) = 562.259, *p* < 0.001; DWV-A: Exp. (*β*) = 2.489, *p* = 0.006; DWV-B: Exp. (*β*) = 4.461, *p* < 0.001; electronic supplementary material, table S2). BQCV killed honeybees the fastest, followed by DWV-B and DWV-A (electronic supplementary material, table S2). Injected virus grew to *ca* 3 × 10^13^ viral genome equivalents at 10 d.p.i. (mean genome equivalents per bee ± s.e.m.: BQCV, 2.39 × 10^13^ ± 8.96 × 10^12^; DWV-A, 3.70 × 10^13^ ± 9.35 × 10^12^; DWV-B, 3.85 × 10^13^ ± 4.81 × 10^12^; electronic supplementary material, figure S4b). Honeybees suffered a slight background infection with DWV-B. However, all viral inocula were devoid of contaminating virus (electronic supplementary material, figure S1), viable and highly virulent in their original host, *A. mellifera*.

### 3.2. Bumblebees

#### 3.2.1. Bumblebees: fed inoculum, satiated

**Figure 1.**
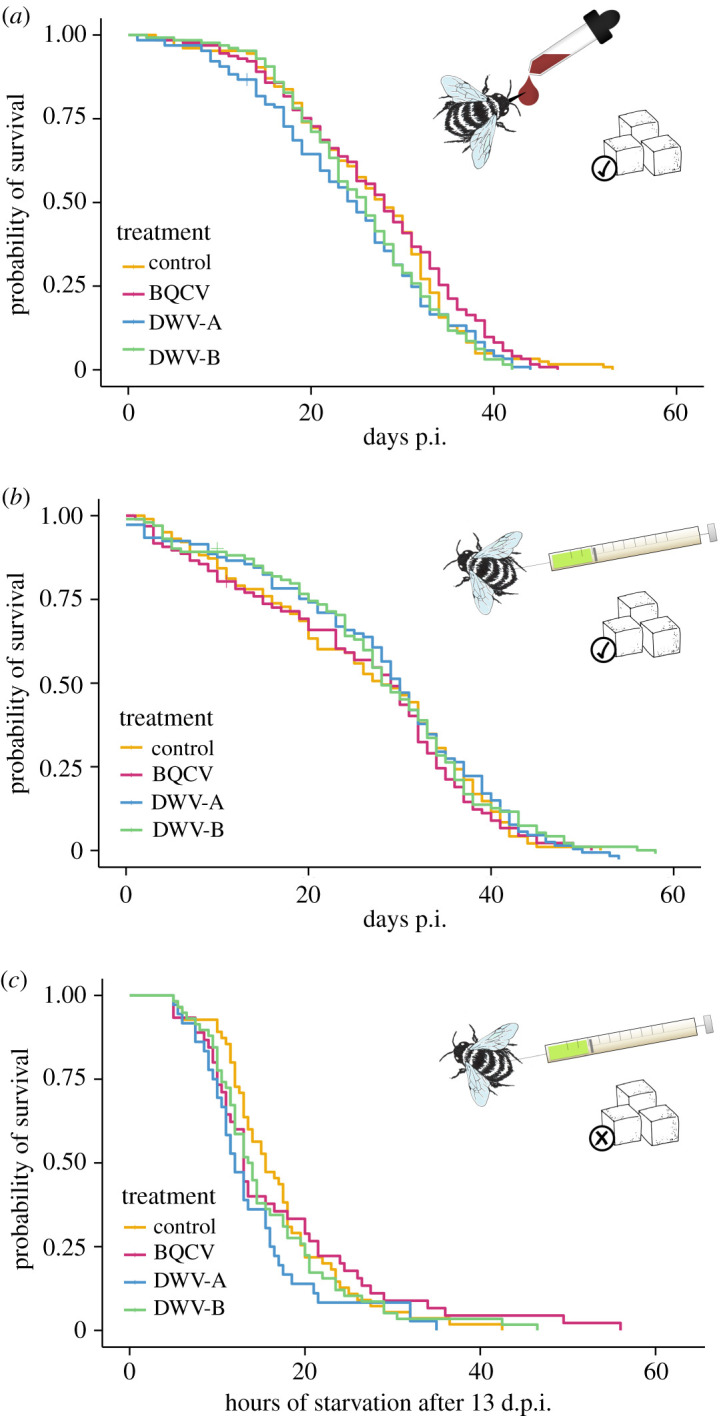
Cox proportional hazards survival curves of bumblebees inoculated with virus. (*a*) Survival in days post-infection (p.i.) of bumblebee workers when inoculated by feeding with 10^9^ viral genome equivalents of BQCV, DWV-A or DWV-B then fed *ad libitum* (*n* = 128 bees per treatment); (*b*) survival in days post-infection (p.i.) of bumblebee workers when inoculated by injection with 10^7^ viral genome equivalents of BQCV, DWV-A or DWV-B then fed *ad libitum* (control, *n* = 102; BQCV, *n* = 97; DWV-A, *n* = 103; DWV-B, *n* = 102); (*c*) survival in hours of bumblebee workers when inoculated by injection with 10^7^ viral genome equivalents of BQCV, DWV-A or DWV-B, fed *ad libitum* for 13 days then starved, defined as hour 0 (control, *n* = 55; BQCV, *n* = 45; DWV-A, *n* = 36; DWV-B, *n* = 58). Symbols represent the method of infection and the availability of sucrose.

*Bombus terrestris* workers inoculated orally and subsequently fed *ad libitum* did not differ in survival compared with controls (Cox proportional hazards: BQCV: Exp. (*β*) = 0.940, *p* = 0.75; DWV-A: Exp. (*β*) = 1.244, *p* = 0.26; DWV-B: Exp. (*β*) = 1.218, *p* = 0.30; figure 1*a*; electronic supplementary material, table S2). Though all viruses were detectable in bumblebee abdomens at 18–25 d.p.i. (figure 1*a*), viral titres were at or just below 10^9^, the amount administered per bumblebee (mean genome equivalents per abdomen ± s.e.m.: BQCV, 1.01 × 10^8^ ± 6.70 × 10^7^; DWV-A, 1.51 × 10^8^ ± 1.37 × 10^8^; DWV-B, 4.42 × 10^10^ ± 3.73 × 10^10^). Bumblebees were devoid of background infection. This experiment suggests that all three viruses can maintain themselves in *B. terrestris* following oral infection, but that they are not virulent when hosts are maintained in the laboratory under benign, satiated conditions.

#### 3.2.2. Bumblebees: injected with inoculum, satiated

**Figure 2.**
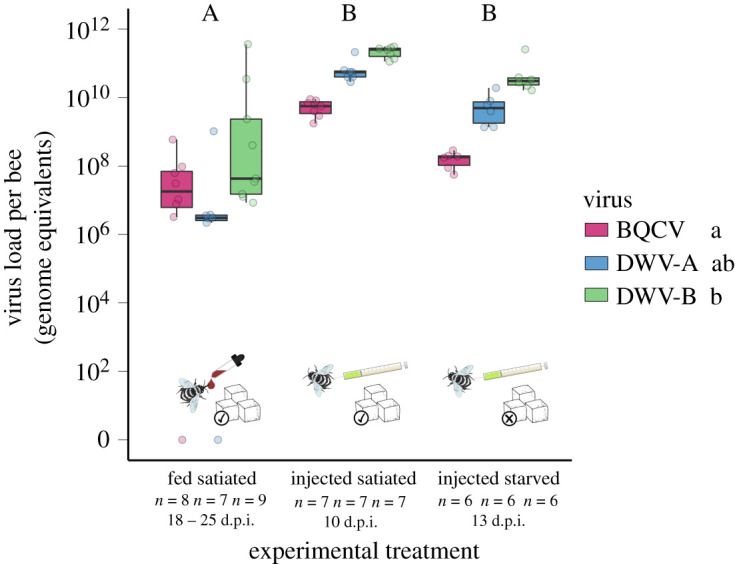
Viral genome equivalents per bumblebee worker abdomen after infection by injection of 10^7^ viral genome equivalents or feeding of 10^9^ viral genome equivalents of BQCV, DWV-A or DWV-B. Across all three viruses, injection resulted in higher viral titres than feeding (horizontal bars: sat.inj–sat.fed *z* = 6.117, *p* ≤ 0.001; starv.inj–sat.fed *z* = 4.096, *p* ≤ 0.001; starv.inj–sat.inj *z* = −1.733, *p* = 0.083). Different uppercase letters indicate significant differences between experimental treatments overall. Across all three experiments, DWV-B titres were significantly higher than BQCV, while DWV-A titre was intermediate and not significantly different from BQCV or DWV-B (vertical bar: DWV-A–DWV-B *z* = 1.005, *p* = 0.315; DWV-B–BQCV *z* = 2.826, *p* = 0.013*; DWV-A–BQCV *z* = 1.776, *p* = 0.076); virus treatments followed by a different lower case letter, *p* < 0.05. Symbols represent the method of infection and the availability of sucrose.

In contrast with honeybees, bumblebees injected with viral inocula and fed *ad libitum* did not die any faster than controls (Cox proportional hazards BQCV: Exp. (*β*) = 0.623, *p* = 0.13; DWV-A: Exp. (*β*) = 1.240, *p* = 0.47; DWV-B: Exp. (*β*) = 0.923, *p* = 0.79; figure 1*b*; electronic supplementary material, table S2). Virus did, though, replicate very well in *B. terrestris* hosts (mean genome equivalents per abdomen ± s.e.m.: BQCV, 5.51 × 10^9^ ± 9.57 × 10^8^; DWV-A, 7.10 × 10^10^ ± 2.21 × 10^10^; DWV-B, 2.21 × 10^11^ ± 2.65 × 10^10^; figure 2). Bumblebees suffered a slight background infection with DWV-B (electronic supplementary material, figure S5). These results indicate that *B. terrestris* workers are competent hosts of BQCV, DWV-A and DWV-B, though these viruses seem not to impact host longevity under benign (satiated) laboratory conditions.

#### 3.2.3. Bumblebees: injected with inoculum, starved

When inoculated by injection and then starved from 13 d.p.i., viral treatment had again no effect on *B. terrestris* mortality (figure 1*c*). When all treatments were analysed simultaneously through to the death of all bumblebees, statistically significant differences among control or treatments were not seen (Cox proportional hazards BQCV: Exp. (*β*) = 1.059, *p* = 0.87; DWV-A: Exp. (*β*) = 1.589, *p* = 0.10; DWV-B: Exp. (*β*) = 1.167, *p* = 0.57; electronic supplementary material, table S2). However, DWV-A inoculated bees exhibited a subtly shorter lifespan (figure 1*c*), dying *ca* 1.6-fold faster than controls, suggesting that DWV-A (but neither DWV-B nor BQCV) might subtly impact *B. terrestris* longevity (see electronic supplementary material, figure S7).

Though smaller worker bumblebees lived longer than larger workers (Cox proportional hazards: Exp. (*β*) = 1.665, *p* = 0.03), bee size did not differ between treatments (electronic supplementary material, figure S6) and bumblebee size did not differentially impact mortality across treatments (electronic supplementary material, table S2).

Viral titres in inoculated bumblebees at 13 d.p.i. where higher for all three viruses than the dose of virus administered: 10^7^ viral genome equivalents (mean per abdomen ± s.e.m.: BQCV 1.67 × 10^8^ ± 3.10 × 10^7^; DWV-A, 6.70 × 10^9^ ± 2.50 × 10^9^; DWV-B, 6.58 × 10^10^ ± 3.47 × 10^10^; figure 2). Bumblebees were not contaminated with other virus (electronic supplementary material, figure S5). This experiment confirms that all three viruses can replicate within *B. terrestris*, and that virus did not markedly shorten bumblebee worker lifespan under food deprivation.

### 3.3. Viral titres across experiments

All three viruses replicated to higher titres in *A. mellifera* than *B. terrestris*. Inoculation of honeybees by injection led to three orders of magnitude higher viral titre (*ca* 3 × 10^13^ viral genome equivalents per bee at 10 d.p.i.) than the equivalent inoculation by injection of bumblebees (*ca* 4 × 10^10^ viral genome equivalents per abdomen at 10 d.p.i.), for all three viruses (figure 2; electronic supplementary material, figure S4b).

Bumblebee inoculation by injection led to higher viral titres than by oral inoculation (figure 2), despite variation in dose (dose injected: 10^7^; dose fed: 10^9^) and duration of infection across experiments (injected, duration of viral replication: 10 d.p.i. and 18–25 d.p.i.; fed, duration of viral replication: 13 d.p.i.). Notably, inoculation with DWV-B led to a significantly higher viral titre than with BQCV within each experiment with bumblebees (figure 2), whereas DWV-A titre lay below BQCV or between DWV-B and BQCV, though not significantly different from either (figure 2).

## 4. Discussion

Here, we show that *B. terrestris* is a competent host for BQCV, DWV-A and DWV-B, suggesting that spillover from honeybees is a potential threat for this and probably other wild bee species. We did not, though, observe impacts of these viruses on bumblebee mortality under laboratory conditions. Furthermore, all three viruses replicated to higher titres in honeybees than in bumblebees, which is surprising, given that honeybees are generally smaller than bumblebees. The higher viral titre per honeybee suggests that these viruses are locally adapted to *A. mellifera*, which is probably their reservoir host.

BQCV, DWV-A and DWV-B have been frequently detected in bumblebees (*Bombus* spp.; [19,24,26,27,29,39,40] and other wild bee species collected from the field (reviewed in [25]), as well as in other insect species associated with honeybees or the flowers they visit (e.g. [54–58]). Moreover, the negative strand of these (+)ssRNA viruses has also been detected in *Bombus* spp. and other wild bee species [19,26–28] as evidence that virus is actively replicating inside these non-*Apis* hosts. Here, we have been able to show unequivocally that all three viruses can replicate to high titres in *B. terrestris*. Additional studies on other non-*Apis* bees, including non-commercial *B. terrestris*, as well as with other honeybee viruses are needed to understand the extent of their host tropism across wild bee species. It will also be important to determine how virulence evolves after a viral jump to a new wild bee species as this is central to disease emergence in the new host [59].

Under benign conditions of the laboratory, we found that BQCV, DWV-A and DWV-B were not virulent (i.e. did not reduce host fitness, *sensu* [60]). Gusachenko *et al*. [61] have recently reported similar findings for DWV-A and DWV-B. These results are surprising because DWV has been associated with field-collected *Bombus* spp. exhibiting clinical symptoms (deformed wings; [23]), which is typical of honeybees when infected by DWV in the pupal stage [47]. Also, when fed [19] or injected [45] into *B. terrestris* workers, DWV has been previously shown to reduce *B. terrestris* lifespan. Differences between former studies and ours may reflect the genetic background of the host; Fürst *et al*. [19] and Graystock *et al*. [45] employed *B. terrestris* from a different commercial source to our study (though [61] used the same source as [20]). Alternatively, it may reflect the source of virus; Fürst *et al*. [19] used a mixed DWV-A/DWV-B inoculum and Graystock *et al*. [45] used DWV isolated from *B. terrestris*, whereas we used DWV-A and DWV-B isolated from *A. mellifera*. Recombination between DWV-A and DWV-B deserves greater attention as a source of virulent virus that may impact both honeybees and bumblebees [44], as does the extent of local adaptation of DWV to a host species.

Another facet of virulence may be the size of the host in relation to viral titre. Honeybee workers are generally smaller than those of bumblebees and, in our experiments, we inoculated each host species with the same viral titre. A direct relationship between host size and inoculum titre could, therefore, account for the higher mortality of honeybees versus bumblebees that we observed. However, viral titres were actually higher in honeybees than bumblebees, arguing against a relationship between host size and inoculum titre that is constant across host bee species. Furthermore, viral titre seems to asymptote after several days in each host species, high in honeybees [47, this study] and lower in bumblebees (electronic supplementary material, figure S2), suggesting that initial viral inoculum size is not related to ensuing viral titre in a host. The relationship between viral titre and host mortality nevertheless deserves greater attention, not only within but also across host species.

Not even under stressful, starvation conditions did we detect a marked effect of either BQCV, DWV-A or DWV-B in reducing *B. terrestris* longevity in the laboratory. Condition-dependent virulence of honeybee viruses in *Bombus* spp. hosts has been seen for slow bee paralysis virus infecting *B. terrestris*, in which longevity was compromised only when hosts were starved [62], and for other bumblebee pathogens such as *Crithidia bombi* [63,64]. We, therefore, urge caution in the interpretation of our result that viral virulence was non-existent in *B. terrestris*. Laboratory conditions may underestimate the impact of honeybee virus spilling over into wild bees in the field, where hosts may be exposed to far harsher environmental conditions and limited resources, e.g. [65]. Insecticides have been highlighted as playing a role in insect, including *Bombus* spp., decline [6,11], with sublethal impacts of novel classes of insecticide on colony fitness [66,67]. Sublethal doses of insecticide can interact with pathogens to elevate host honeybee mortality [68–70], and may represent another condition-dependent factor for bumblebees and other wild bee species that exacerbates the impact on them of viral spillover from honeybees. Field-realistic experimental paradigms are now needed to reveal the role of viral spillover for the individual, colony and population fitness of wild bee species as well as additional experiments examining other response variables than mere mortality, e.g. offspring production, pupal development and foraging efficiency. Changes in sublethal parameters like these could decrease the success of a social bee colony enormously. Furthermore, our non-benign scenario (starvation) may have been too stressful to allow expression of condition-dependent virulence; use of more natural levels of stress, as may be typically experienced by bees in the field, is warranted to reveal condition-dependent virulence.

We found that viral titres were lower and the impact on host mortality was non-existent when BQCV, DWV-A or DWV-B was injected into *B. terrestris* versus injected into *A. mellifera*. These results suggest that virus may be locally adapted to its host, and that *A. mellifera* may be the reservoir host for all three viruses. The immediate impact of viral spillover from honeybees to bumblebees and other wild bee species might then indeed be low, as we found under our benign laboratory conditions. But transmission from bumblebee to bumblebee could lead to local adaption of a virus to a *Bombus* host, with unknown consequences of pathogen spill-back from bumblebees and other wild bee species to honeybees if viral adaptation to the novel host (*Bombus*) trades off with a loss of virulence in the original host (*Apis*) [71–73]. The speed with which local adaptation to a novel host occurs, its relationship to virulence and whether it results in a loss of viral fitness or virulence in the reservoir host will help determine the impact of viral pathogen spillover for the entire bee pollinator community [74].

It is unsurprising that we found inoculation by injection to lead to higher viral titres than by oral inoculation of bumblebees. Injection of a pathogen into the insect haemocoel gives the pathogen access to the entire host body tissue, whereas oral infection initially gives it access to the gut alone. The former route of transmission, injection into the haemocoel, through *V. destructor* host feeding is thought to account for the huge increase in viral prevalence and intensity of infection of DWV in honeybees [75,76]. In support of this view, injection of another honeybee virus, Israeli acute paralysis virus, into *B. terrestris* led to systemic infection and rapid host death, whereas oral infection led to infection of the host gut in a dose-dependent manner and with more limited impact on host health [49].

That BQCV was extremely virulent in our honeybee assay is at first sight surprising because BQCV is widespread and highly prevalent in honeybee populations [24,26,29,30,77]. Both Retschnig *et al*. [78] and Doublet *et al*. [70] found no effect of feeding BQCV on adult honeybee mortality, suggesting it is a benign pathogen, though lethal when fed to queen [79], drone [80] and worker [70] pupae. The high virulence of BQCV in honeybees that we here and others [81] have observed is probably due to it having been injected into hosts. From epidemiological theory, pathogen prevalence is often inversely related to virulence in insect host populations [82]. To explain its high prevalence in honeybees, we suggest that BQCV is rather benign when infecting adult *A. mellifera* workers through its typical faecal–oral route of transmission.

The Western honeybee is the dominant flower visitor across most terrestrial ecosystems of the world [83]. Dominant species in a community often disproportionately influence pathogen transmission and dynamics [84] through their central role in contact networks [85], exacerbated in the case of *A. mellifera* because it is probably the reservoir host of BQCV, DWV-A, DWV-B. Though we recorded little to no virulence of these viruses on *B. terrestris* under laboratory conditions, their impact on this and other bee species (and other flower visitors) under field-realistic conditions should be the focus of future studies to evaluate the role of viral spillover in wild bee decline.

## Supplementary Material

Supplementary Materials

Reviewer comments

## Data accessibility

All data supporting the findings of this study are available from the Dryad Data Repository: https://doi.org/doi:10.5061/dryad.fxpnvx0nt [86].
