# Supplementary material for: Experimental infection of bumblebees with honeybee-associated viruses: no direct fitness costs but potential future threats to novel wild bee hosts
Source: R Soc Open Sci. 2020 Jul 8;7(7):200480. doi: 10.1098/rsos.200480 (PMC7428241; doi:10.1098/rsos.200480)
Supplement: Supplementary Materials [file rsos200480supp1.docx]

**Supplementary Materials**

Article title: Experimental viral spill-over from honey bees to bumble bees: no immediate fitness costs but potential future threats to novel wild bee hosts

Authors: Anja Tehel^1,*^, Tabea Streicher^1^, Simon Tragust^1^, and Robert J. Paxton^1,2^

Addresses:

^1^General Zoology, Institute for Biology, Martin Luther University Halle-Wittenberg, Hoher Weg 8, 06120 Halle (Saale), Germany

^2^German Centre for Integrative Biodiversity Research (iDiv) Halle-Jena-Leipzig, Deutscher Platz 5e, 04103 Leipzig, Germany

Contents:

Supplementary Methods: Additional information to molecular biological (qPCR) and experimental methods used in this study

Figure S1: Viral composition of inocula used in all experiments

Figure S2: Virus load of two different data sets of bumble bees fed with DWV-A and DWV-B

Figure S3: Proportion of bees exhibiting arbitrary movements during starvation experiment

Figure S4: Survival and virus load of honey bee workers when injected with DWV-A, DWV-B and BQCV

Figure S5: Viral load of bumble bee workers of all four treatments of the three inoculation experiments

Figure S6: Bumble bee size distribution across treatments in the starvation experiment

Figure S7: Survival of DWV-A injected bumble bee workers under starvation conditions in more detail

Table S1: Viral presence in bumble bee and honey bee colonies used as sources of bees for experiments

Table S2: Cox proportional hazards models of worker bee mortality following experimental infection

**Supplemental Methods: Additional information to molecular biological (qPCR) and experimental methods used in this study.**

**Quantification of viral titre**

Most known viruses of honey bees are positive single stranded (+)ssRNA viruses [1,2], a class of virus know to have extremely high rates of mutation [3]. DWV, a (+)ssRNA virus, is a picorna-like virus in the family *Iflaviridae* that often leads to crippled wings and high mortality in honey bees; it is efficiently vectored by the mite *V. destructor* [2,4–8]. It comprises at least three distinct genotypes [9,10], two of which (DWV-A and DWV-B) are widespread and differentially virulent in adult honey bees [11] though not in honey bee pupae [5]. BQCV, another (+)ssRNA virus (family Dicistroviridae), kills honey bee queen pupae, leaving them mottled black [12], but is frequently found in *A. mellifera* workers [13]. Together with DWV, it is one of the most prevalent viruses detected in non-*Apis* bees [13–15].

To check that bumble bees (12 source colonies: labelled B1 to B12) and honey bees (2 source colonies, labelled 5.1 and G) as well as the fresh-frozen pollen pellets were devoid of viral pathogens, we tested them by real-time quantitative PCR (qPCR) for seven common honey bee viral targets: DWV-A, DWV-B, BQCV, *Acute bee paralysis virus* (ABPV), *Chronic bee paralysis virus* (CBPV), *Sacbrood virus* (SBV), and *Slow bee paralysis virus* (SBPV) using primers given in [13], Supplementary Table S1 for colonies). We additionally screened colonies for the Microsporidia *Nosema apis*, *Nosema bombi* and *Nosema ceranae* using methods in Fries *et al*. [16]; all PCRs were negative, suggesting no background microsporidial infection.

For viral screening, we collected 10-20 adult worker bees per colony or 2 x 0.3 g of pollen pellets (i.e. two samples), crushed them in a plastic RNAse-free mesh bag (BioReba, Reinach, Switzerland) with ultrapure diethylpyrocarbonate (DEPC)-treated water (500µl per honey bee, 1000µl per bumble bee, 1000 µl per 0.3 g of pollen) after snap-freezing them on dry ice, and then recovered 100 µL of homogenate from beyond the BioReba mesh for RNA extraction.

We ran quality control checks on each 96-well qPCR reaction plate. To check that the correct template had been amplified, PCR products were denatured for one minute at 95 °C, cooled to 55 °C for one minute, and then a melting profile was generated from 55 °C to 95 °C (0.5 °C per second increment) to ensure the expected product had been amplified, namely a single peak at the product’s dissociation temperature (Tm). In addition, a virus-free (negative control) and a virus-infected (positive control) sample were included on each 96-well plate. A host housekeeping gene, *Apis mellifera*-β-actin, was also amplified for all samples as an internal reference marker using the primers given in Locke *et al.* [17]. All PCRs showed the expected signals for the negative (no signal) and positive (Ct < 35) controls and β-actin gave Ct values for all samples between 16 and 26, suggesting no contamination or error in pipetting, no RNA degradation, no error in RNA extraction, and no failure in cDNA synthesis.

**Experimental inoculation**

*Bumble bees – fed inoculum, satiated.*

Freshly emerged (24-48 after eclosion) bumble bee workers, collected as described in 2.3 “Bumble bees general handling”, were transferred individually to an inverted plastic cup on a plastic lid (one bee per cup) then starved for 4 - 5 hours. Thereafter, bees were individually fed with 10^9^ viral genome equivalents (or the equivalent control solution devoid of virus) in 10 µL of 50% (w/v) sucrose solution pipetted to the bottom of the cup. Food intake was observed; bees that did not consume the entire inoculum within 15 min were excluded from the experiment. After an additional 1 – 2 h to ensure that bees did not regurgitate food, they were transferred to a new, autoclaved metal cage in small groups (5 to 10 bees per cage grouped according to treatment).

**Supplementary Figure S1: Viral composition of inocula used in all experiments**

**
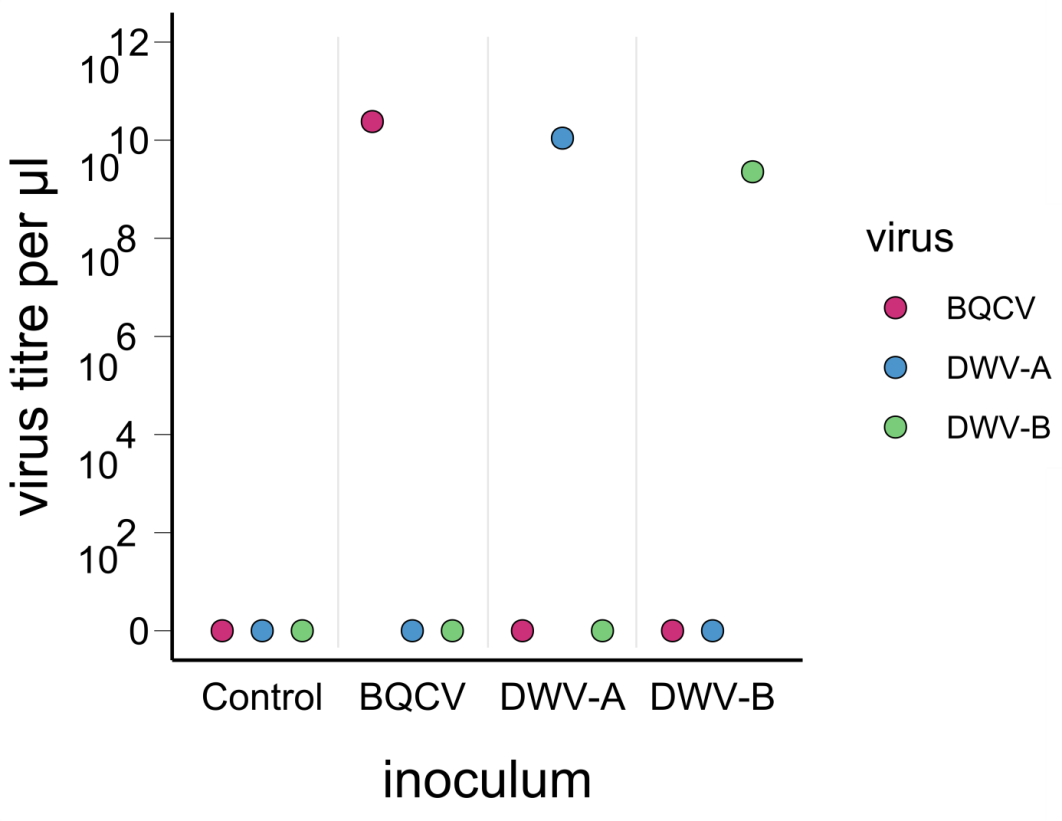
**

**Figure S1.** Viral titre of 1 µl of the inocula that were used for all experiments. All inocula were screened for the presence of DWV-A, DWV-B and BQCV as well as ABPV, CBPV, SBPV and SBV; contaminant virus was not detected. To propagate DWV-A and DWV-B for experimental inocula, we used the inocula from Tehel *et al.* [5], a propagation from the genotype-specific inocula of McMahon *et al*. [11], which had originally been extracted from an adult, heavily virus-infected honey bee with normal wings from Great Britain (DWV-A) or Germany (DWV-B). Our BQCV inoculum was prepared by propagating the BQCV inoculum of Doublet *et al*. [18], originally derived from infected *A. mellifera* collected in Harpenden, England [12]. Viral propagation in honey bee pupae followed precisely methods in Tehel *et al.* [5]. We crushed three honey bee pupae in 500 µL of 0.5 M of potassium phosphate buffer (PPB) (pH 8.0) using a plastic pestle, 50 µL of which was used for RNA isolation. Viral detection by qPCR followed methods described above. We always generated the correct virus inoculum from the original inoculum, which was devoid of other viruses. Batches of pupal homogenates cross-contaminated with another virus (either BQCV or DWV-B were occasional cross-contaminants) were not used as inocula. Un-injected white-eyed pupae devoid of virus by qPCR were used to generate a control inoculum that was identical to viral inocula, but for the lack of virus. Absolute quantification of virus in inocula followed methods in Tehel *et al*. [5] using a dilution series (10^−1^–10^−8^) of an external DNA standard to generate calibration curves. Duplicate qPCRs were run for each sample and the mean Ct value was used; Ct values always differed by <0.5 between duplicates. Primer efficiencies were 96 % for DWV-A, 95 % for a DWV-B and 98 % for BQCV, with correlation coefficients (R^2^) ≥ 0.9.

**Supplementary Figure S2: Virus load of two different data sets of bumble bees fed with DWV-A and DWV-B**


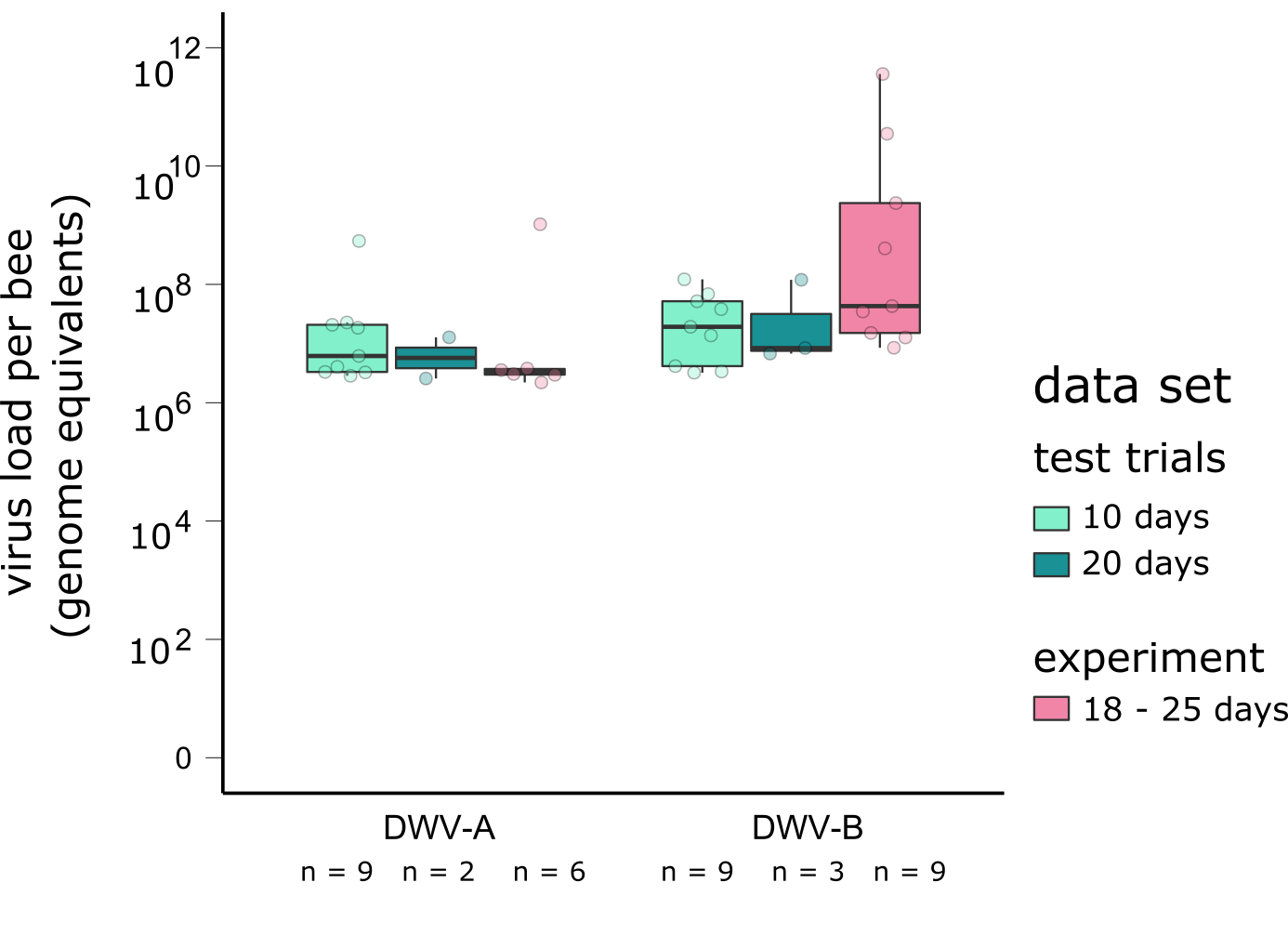


**Figure S2.** Viral genome equivalents in bumble bee worker abdomens after feeding with 10^9^ viral genome equivalents of DWV-A or DWV-B in initial trials, then freeze killed at 10 or 20 days post infection (d.p.i.); in the reported starvation experiment, bees were freeze killed 18-25 d.p.i.. Viral titres did not differ across treatments and experiments (LM: DWV-A F_2,14_ = 0.135, p =0.875; DWV-B F_2,18_ = 2.727, p = 0.092). Two bees were excluded (one DWV-A inoculated bee in the test trial at 20 d.p.i.; one DWV-A inoculated bee in the reported experiment) because inoculation failed (the bee was devoid of virus).

**Supplementary Figure S3: Proportion of bees exhibiting arbitrary movements during the starvation experiment**


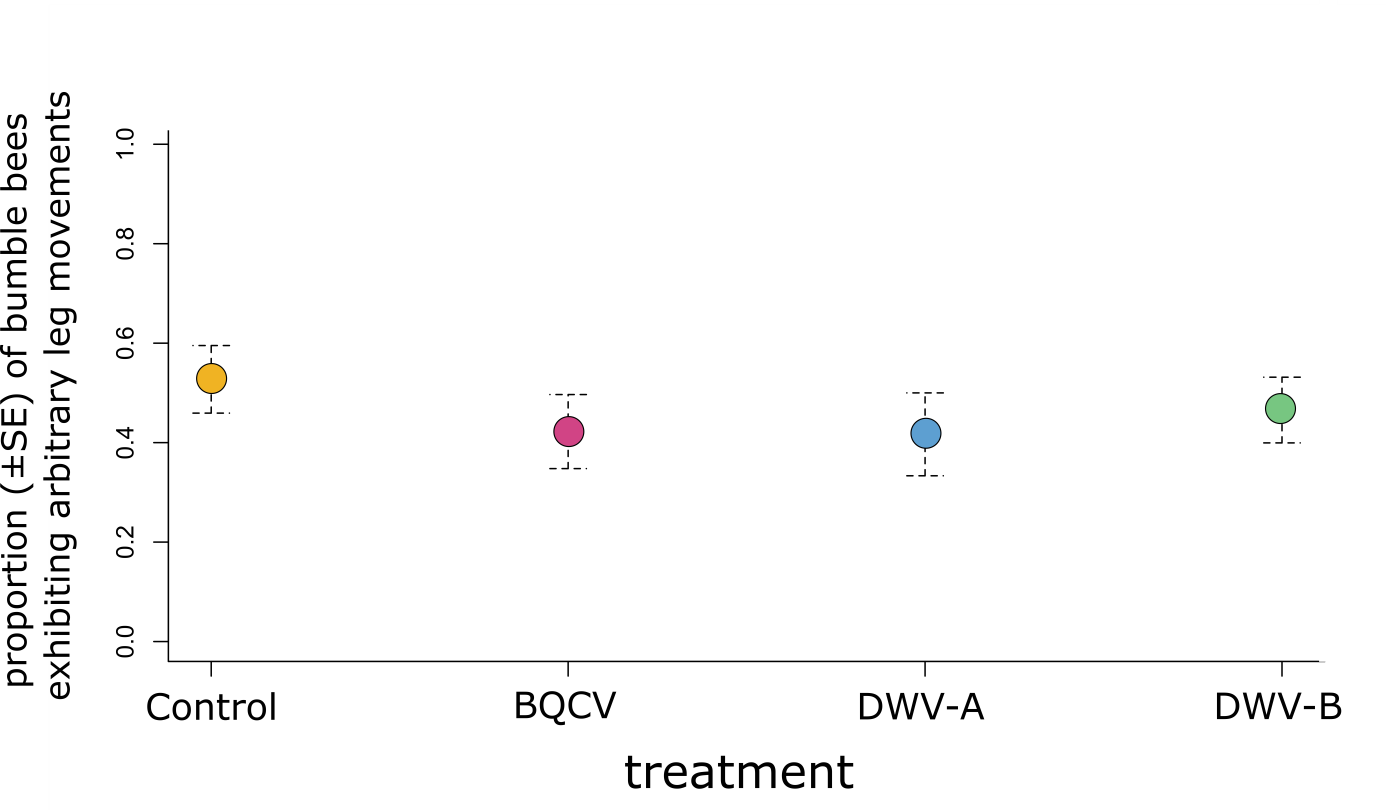


**Figure S3.** Proportion of bees lying on their backs and exhibiting arbitrary movements with their legs when starved during the starvation experiment. Time of death was difficult to determine because, after 10-40 hours, bees fell on their backs and arbitrarily waved their legs. We therefore measured the time when bees first fell on their backs as well as 2 hours after continuously remaining on their backs, waiving legs arbitrarily. Arbitrary leg movement was independent of treatment. Moreover, use of the first time point (first time on the back) versus the second time point (continuously on back for over two consecutive hours) made no qualitative difference to statistical analyses and so results from only the first time point are reported. Treatments did not differ significantly from each other (LM: F_3,190_ = 0.502, p = 0.681).

**Supplementary Figure S4: Survival and virus load of honey bee workers when injected with DWV-A, DWV-B and BQCV**


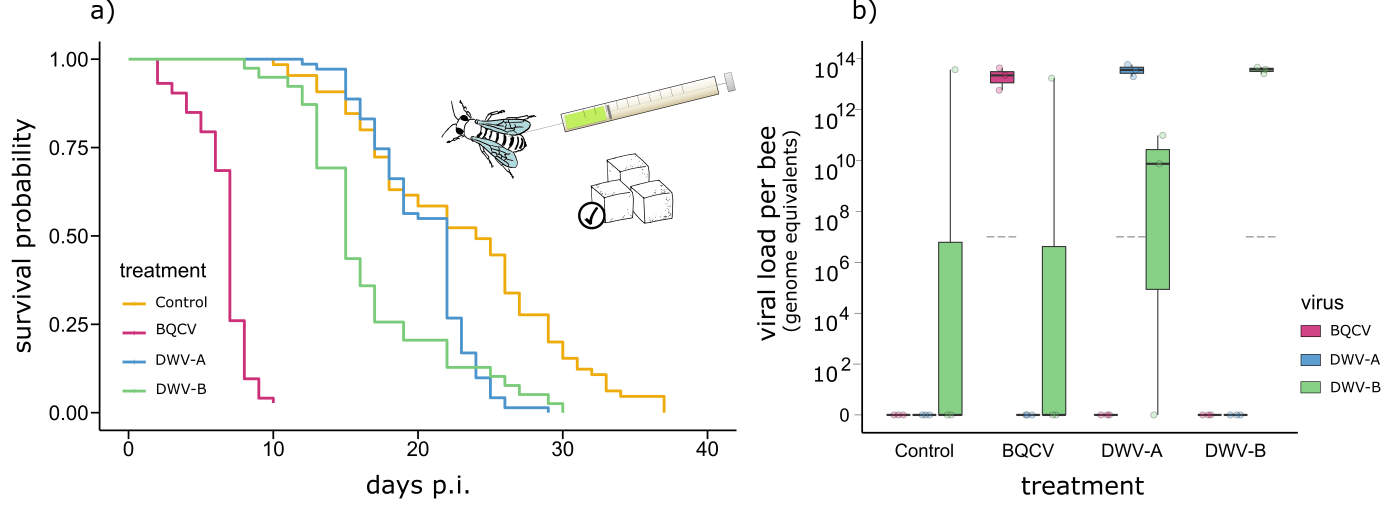


a

b

b

c

**Figure S4.** (a) Survival in days post infection (p.i.) of honey bee workers when inoculated by injection with 10^7^ viral genome equivalents of BQCV, DWV-A or DWV-B then fed *ad libitum* (Cox proportional hazards survival curves: Control, n = 66; BQCV, n = 73; DWV-A, n = 71; DWV-B n = 39); virus treatments followed by a different lower case letter, P<0.05 (details in Supplementary Table S2); (b) viral titre per honey bee at 10 days p.i. (box and whiskers plot of n = 3 bees per treatment, dashed lines represent viral genome equivalents initially injected per bee); one honey bee source colony had a background infection of DWV-B (see Supplementary Material S1), hence the background infection of DWV-B in the control and across all treatments. To obtain freshly emerged bees for the experiment, frames of sealed brood from our two honey bee colonies were kept in an incubator (35°C) overnight and, the next morning, the freshly eclosed workers were collected.

**Supplementary Figure S5: Viral load of bumble bee workers of all four treatments of the three inoculation experiments**

**
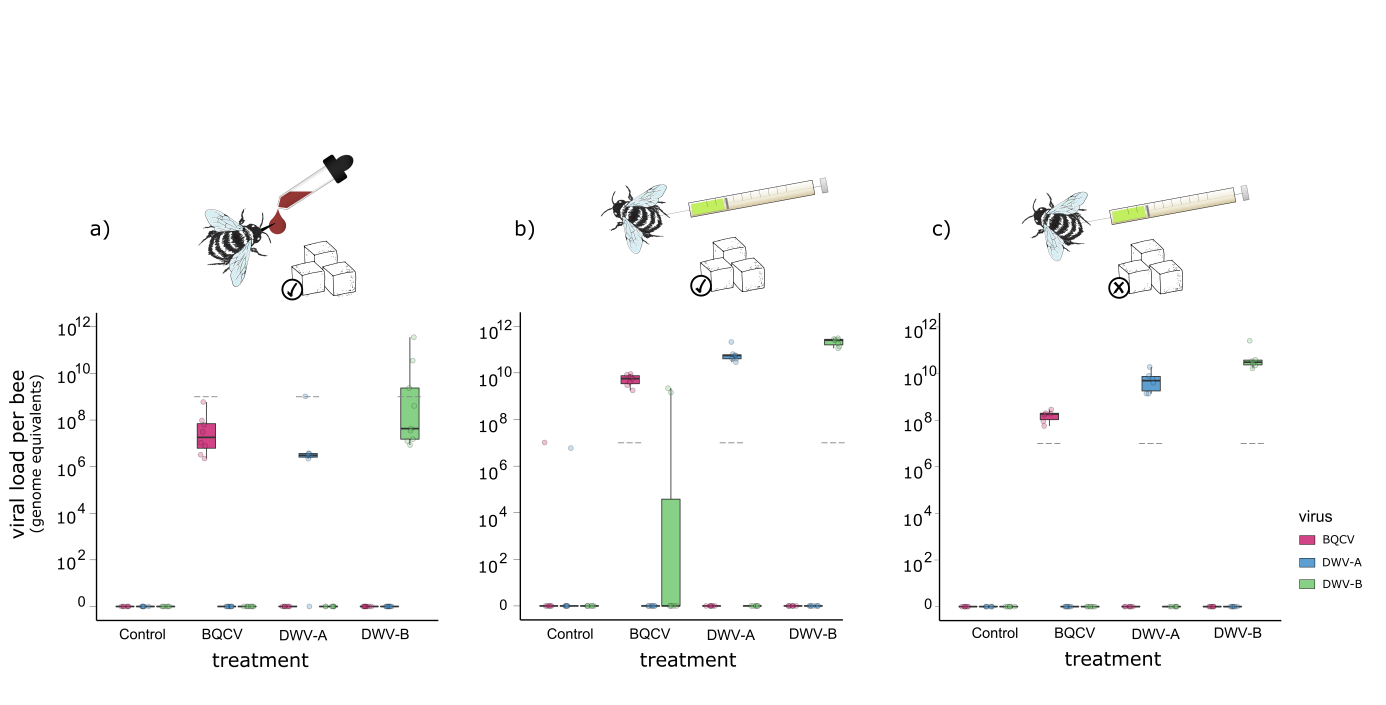
**

**Figure S5.** Viral titre of bumble bee workers (a) when inoculated by feeding with 10^9^ viral genome equivalents of BQCV, DWV-A or DWV-B then fed *ad libitum;* values per bumble bee abdomen at 18-25 days p.i. (box and whiskers plot of n = 7 bees per treatment, dashed lines represent viral genome equivalents initially fed per bee); (b) when inoculated by injection with 10^7^ viral genome equivalents of BQCV, DWV-A or DWV-; values per bumble bee abdomen at 10 days p.i. (box and whiskers plot of n = 6, 8, 9 and 7 bees per respective treatment, dashed lines represent viral genome equivalents initially injected per bee); one bumble bee source colony had a background infection of DWV-B (see Supplementary Material S1); (c) when inoculated by injection with 10^7^ viral genome equivalents of BQCV, DWV-A or DWV-B, fed *ad libitum* for 13 days then starved at hour 0; values per bumble bee abdomen at 13 days post infection (box and whiskers plot of n = 6, bees per treatment, dashed lines represent viral genome equivalents initially injected per bee). These data for are reported in Figure 2 of the main manuscript only for the virus used in each treatment. Here we show background contamination with other viruses, which was negligible across experiments. Symbols are representing the way of infection and the availability of food in form of sugar.

**Supplementary Figure S6: Bumble bee size distribution across treatments in the starvation experiment**


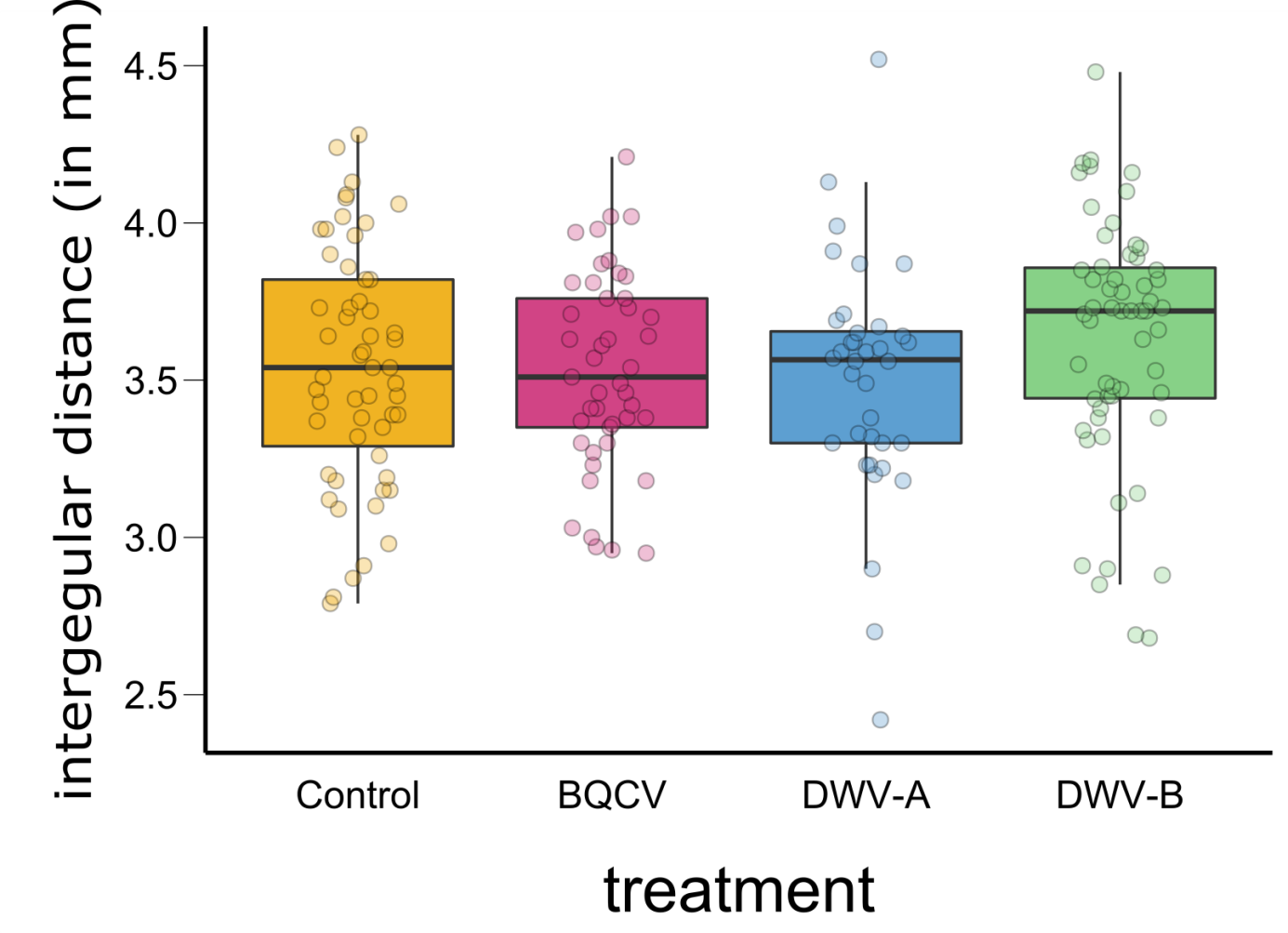


**Figure S6.** Distribution of bumble bee intertegular distances in mm (a measure of body size) across treatments within the starvation experiment. Treatments did not differ significantly from each other (LM: F_3,190_ = 1.172, p = 0.322). Additionally bees were weighed directly after death. As weight and intertegular distance were highly correlated (Spearman rank correlation rho = 0.762, p <0.001), intertegular distance alone was used in further analyses.

**Supplementary Figure S7: Survival of DWV-A injected bumble bee workers under starvation conditions in more detail**

**
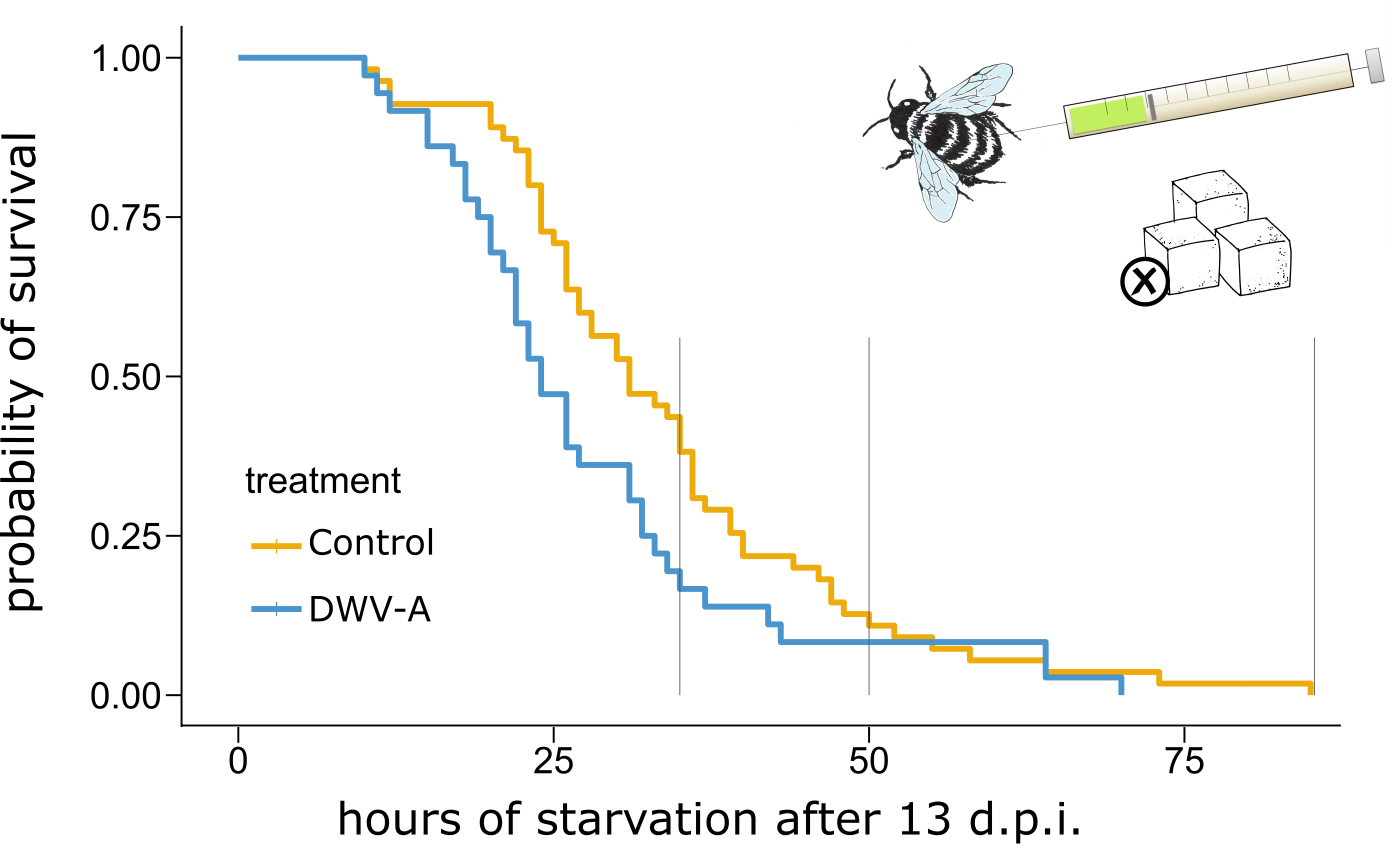
**

**Figure S7.** DWV-A virulence when injected into virus-free, 24-48 h old bumble bee workers that were fed *ad libitum* till day 13 p.i. then kept without food in the starvation experiment. Fitted Cox proportional hazards survival curves in hours post-initiation of starvation till death of all individuals. Vertical lines represent time points where significance was tested using a Cox proportional hazards model (after 35h and death of 70% of bumble bees, Exp. (β) = 2.192, p = 0.016; after 50h when the lifespans of treatment and control nearly converge (see Fig. 1 of Fürst et al. 2014) and death of 90% of bumble bees, Exp. (β) = 1.808, p = 0.050; and at the end of the experiment, after death of all bumble bees, Exp. (β) = 1.725, P = 0.060; treatment DWV-A, n = 36 bumble bees; Control n = 55 bumble bees). DWV-B and BQCV (see Fig. 1c) did not cause significantly shorter lifespan when checked at the same time points (35 h after initiation of starvation: Cox proportional hazards for DWV-B: Exp. (β) = 1.332, p = 0.230; BQCV: Exp. (β) = 1.441, p = 0.260; 50 h after initiation of starvation: Cox proportional hazards for DWV-B: Exp. (β) = 1.191, p = 0.380; BQCV: Exp. (β) = 1.098, p = 0.750).

**Supplementary Table S1: Viral presence in bumble bee and honey bee colonies used as sources of bees for experiments**

**Table S1** Viral presence in 12 bumble bee and 2 honey bees colonies used as sources of bees for experiments; qPCR Ct values for DWV-A, DWV-B, BQCV, *Sac brood virus* (SBV), *Chronic bee paralysis virus* (CBPV) and *Slow bee paralysis virus* (SBPV); x: Ct value > 40; n.t.: not tested. one *B. terrestris* colony had a Ct value of 27 for DWV-B and three had a Ct value of 33 - 35 for BQCV, suggesting slight background infection while both honey bee colonies and both pollen samples were devoid of virus prior to experimentation

| Experiment | date | colony | DWV-A | DWV-B | BQCV | SBV | CBPV | SBPV |
| --- | --- | --- | --- | --- | --- | --- | --- | --- |
| Bombus fed.sat. | 06.03.2018 | B1 | x | x | 37 | n.t. | n.t. | n.t. |
|  |  | B2 | x | x | 38 | n.t. | n.t. | n.t. |
|  |  | B3 | x | x | 38 | n.t. | n.t. | n.t. |
|  |  | B4 | 27 | x | 39 | n.t. | n.t. | n.t. |
|  |  | B5 | x | x | 37 | n.t. | n.t. | n.t. |
| Bombus inj.sat. | 31.05.2018 | B6 | x | x | 37 | x | x | x |
|  |  | B7 | x | x | 37 | x | x | x |
|  |  | B8 | x | x | 35 | x | x | x |
|  |  | B9 | x | x | 33 | x | x | x |
| Bombus inj.starv. | 18.01.2019 | B10 | x | x | 36 | x | n.t. | n.t. |
|  |  | B11 | x | x | 37 | x | n.t. | n.t. |
|  |  | B12 | x | x | 34 | x | n.t. | n.t. |
|  | **date** | **colony** | **DWV-A** | **DWV-B** | **BQCV** | **SBV** | **CBPV** | **SBPV** |
| Apis inj.sat. | 14.06.2018 | G | x | 27 | 38 | x | x | x |
|  |  | 5.1 | 27 | 33 | 36 | 36 | x | x |
|  | 28.06.2018 | G | x | x | x | x | x | x |
|  |  | 5.1 | x | x | x | x | x | x |
|  | 25.07.2018 | G | x | x | x | x | x | x |
|  |  | 5.1 | x | x | x | x | x | x |
|  | 03.08.2018 | G | x | 36 | x | x | x | x |
|  |  | 5.1 | x | 34 | x | x | x | x |

**Supplementary Table S2: Cox proportional hazards models of worker bee mortality following experimental infection**

**Table S2.** Final Cox proportional hazards model of worker bee mortality following experimental infection (s.e., standard error); within an experiment, p values show significance differences of β (the standardised effect size) of treatment *versus* control whilst different lower case letters show statistical differences among β by a *posteriori* Tukey test with Westfall correction for multiple comparisons; exp (β) is equivalent to the hazard ratio, the instantaneous probability of death (a high value is equivalent to a high probability of death)

|  | **Coefficients** | | |  |  |  | **model testing** | | |
| --- | --- | --- | --- | --- | --- | --- | --- | --- | --- |
|  | **β** | **s.e. (β)** | **exp. (β)** | **z** | **p** |  | **Chi^2^** | **df** | **p** |
| **Honey bees** |  |  |  |  |  |  |  |  |  |
| ***Injection - benign*** |  |  |  |  |  | **treatment** | 46.144 | 3 | <0.001 |
| **Control** | 0 a |  | 1 |  |  |  |  |  |  |
| **BQCV** | 6.331 b | 0.683 | 562.259 | 9.28 | <0.001 |  |  |  |  |
| **DWV-A** | 0.912 c | 0.329 | 2.489 | 2.77 | 0.006 |  |  |  |  |
| **DWV-B** | 1.495 c | 0.351 | 4.461 | 4.26 | <0.001 |  |  |  |  |
|  |  |  |  |  |  |  |  |  |  |
| **Bumble bees** |  |  |  |  |  |  |  |  |  |
| ***Feeding - benign*** |  |  |  |  |  | **treatment** | 3.313 | 3 | 0.371 |
| **Control** | 0 |  | 1 |  |  |  |  |  |  |
| **BQCV** | -0.061 | 0.193 | 0.940 | -0.32 | 0.75 |  |  |  |  |
| **DWV-A** | 0.218 | 0.194 | 1.244 | 1.12 | 0.26 |  |  |  |  |
| **DWV-B** | 0.198 | 0.191 | 1.218 | 1.03 | 0.30 |  |  |  |  |
|  |  |  |  |  |  |  |  |  |  |
| ***Injection - benign*** |  |  |  |  |  | **treatment** | 5.090 | 3 | 0.165 |
| **Control** | 0 |  | 1 |  |  |  |  |  |  |
| **BQCV** | -0.473 | 0.313 | 0.623 | -1.51 | 0.13 |  |  |  |  |
| **DWV-A** | 0.214 | 0.294 | 1.240 | 0.73 | 0.47 |  |  |  |  |
| **DWV-B** | -0.080 | 0.295 | 0.923 | -0.27 | 0.79 |  |  |  |  |
|  |  |  |  |  |  |  |  |  |  |
| ***Injection - starved*** |  |  |  |  |  | **treatment** | 2.880 | 3 | 0.412 |
| **Control** | 0 a |  | 1 |  |  |  |  |  |  |
| **BQCV** | 0.044 a | 0.272 | 1.059 | 0.16 | 0.87 |  |  |  |  |
| **DWV-A** | 0.462 a | 0.282 | 1.589 | 1.64 | 0.10 |  |  |  |  |
| **DWV-B** | 0.141 a | 0.248 | 1.167 | 0.57 | 0.57 |  |  |  |  |
| **Age** | -0.010 a | 0.020 | 0.988 | -0.51 | 0.61 |  |  |  |  |
| **size** | 0.542 b | 0.234 | 1.665 | 2.32 | 0.03 |  |  |  |  |

**References to Suplementary Materials:**

1. Chen YP, Siede R. 2007 Honey bee viruses. *Adv. Virus Res.* **70**, 33–80. (doi:10.1016/S006 5-3527(07)70002-7)

2. Grozinger CM, Flenniken ML. 2019 Bee viruses: ecology , pathogenicity, and impacts. *Annu. Rev. Entomol.* **64**, 205–226. (doi:10.1146/annurev-ento-011118- 111942)

3. Holmes EC. 2009 *The Evolution and Emergence of RNA Viruses*. New York: Oxford University Press Inc. (doi:10.1017/CBO9781107415324.004)

4. de Miranda JR, Genersch E. 2010 Deformed wing virus. *J. Invertebr. Pathol.* **103**, S48–S61. (doi:10.1016/j.jip.2009.06.012)

5. Tehel A, Vu Q, Bigot D, Gogol-Döring A, Koch P, Jenkins C, Doublet V, Theodorou P, Paxton R. 2019 The two prevalent genotypes of an emerging infectious disease, Deformed wing virus, cause equally low pupal mortality and equally high wing deformities in host honey bees. *Viruses* **11**, 114. (doi:10.3390/v11020114)

6. Brettell LE, Mordecai GJ, Schroeder DC, Jones IM, Da Silva JR, Vicente-Rubiano M, Martin SJ. 2017 A comparison of Deformed wing virus in deformed and asymptomatic honey bees. *Insects* **8**. (doi:10.3390/insects8010028)

7. Francis RM, Nielsen SL, Kryger P. 2013 *Varroa*-virus interaction in collapsing honey bee colonies. *PLoS One* **8**, e57540. (doi:10.1371/journal.pone.0057540)

8. Martin SJ, Brettell LE. 2019 Deformed wing virus in honeybees and other insects. *Annu. Rev. Virol.* **6**, 49–69. (doi:10.1146/annurev-virology-092818-015700)

9. Mordecai GJ, Wilfert L, Martin SJ, Jones IM, Schroeder DC. 2016 Diversity in a honey bee pathogen: first report of a third master variant of the Deformed wing virus quasispecies. *Int. Soc. Microb. Ecol.* **10**, 1264–1273. (doi:10.1038/ismej.2015.178)

10. Kevill JL, Highfield A, Mordecai GJ, Martin SJ, Schroeder DC. 2017 ABC assay: method development and application to quantify the role of three DWV master variants in overwinter colony losses of european honey bees. *Viruses* **9**, 314. (doi:10.3390/v9110314)

11. McMahon DP, Natsopoulou ME, Doublet V, Fürst M, Weging S, Brown MJF, Gogol-Döring A, Paxton RJ. 2016 Elevated virulence of an emerging viral genotype as a driver of honeybee loss. *Proc. R. Soc. Biol. Sci.* **283**, 443–449. (doi:10.1098/rspb.2016.0811)

12. Bailey L, Woods RD. 1977 Two more small RNA viruses from honey bees and further observations on *Sacbrood* and *Acute bee-paralysis viruses*. *J. Gen. Virol.* **37**, 175–182.

13. McMahon DP, Fürst MA, Caspar J, Theodorou P, Brown MJF, Paxton RJ. 2015 A sting in the spit: widespread cross-infection of multiple RNA viruses across wild and managed bees. *J. Anim. Ecol.* **84**, 615–624. (doi:10.1111/1365-2656.12345)

14. Murray EA, Burand J, Trikoz N, Schnabel J, Grab H, Danforth BN. 2019 Viral transmission in honey bees and native bees, supported by a global Black queen cell *virus* phylogeny. *Environ. Microbiol.* **21**, 972–983. (doi:10.1111/1462-2920.14501)

15. Radzevičiūtė R, Theodorou P, Husemann M, Japoshvili G, Kirkitadze G, Zhusupbaeva A, Paxton RJ. 2017 Replication of honey bee-associated RNA viruses across multiple bee species in apple orchards of Georgia, Germany and Kyrgyzstan. *J. Invertebr. Pathol.* **146**, 14–23. (doi:10.1016/j.jip.2017.04.002)

16. Fries I, Chauzat M, Chen Y, Doublet V, Genersch E, Gisder S, Higes M, McMahon DP, Natsopoulou M, Paxton RJ *et al.* 2013 Standard methods for *Nosema* research. *J. Apic. Res.* **52**. (doi:10.3896/IBRA.1.52.1.14)

17. Locke B, Forsgren E, Fries I, de Miranda JR. 2012 Acaricide treatment affects viral dynamics in *Varroa destructor*-infested honey bee colonies via both host physiology and mite control. *Appl. Environ. Microbiol.* **78**, 227–235. (doi:10.1128/AEM.06094-11)

18. Doublet V, Labarussias M, de Miranda JR, Moritz RFA, Paxton RJ. 2015 Bees under stress: sublethal doses of a neonicotinoid pesticide and pathogens interact to elevate honey bee mortality across the life cycle. *Environ. Microbiol.* **17**, 969–983. (doi:10.1111/1462-2920.12426)
