# Supplementary material for: Experimental infection of bumblebees with honeybee-associated viruses: no direct fitness costs but potential future threats to novel wild bee hosts
Source: R Soc Open Sci. 2020 Jul 8;7(7):200480. doi: 10.1098/rsos.200480 (PMC7428241; doi:10.1098/rsos.200480)
Supplement: Reviewer comments [file rsos200480_review_history.pdf]

THE ROYAL SOCIETY
PUBLISHING

ROYAL SOCIETY
OPEN SCIENCE

Experimental infection of bumblebees with honeybee-associated viruses: no direct fitness costs but potential future threats to novel wild bee hosts

Anja Tehel, Tabea Streicher, Simon Tragust and Robert J. Paxton

Article citation details
R. Soc. open sci. **7**: 200480.
http://dx.doi.org/10.1098/rsos.200480

Review timeline

Original submission:	24 March 2020
Revised submission:	5 June 2020
Final acceptance:	10 June 2020

Note: Reports are unedited and appear as submitted by the referee. The review history appears in chronological order.

Note: This manuscript was transferred from another Royal Society journal without peer review.

Review History

RSOS-200480.R0 (Original submission)

Review form: Reviewer 1

Is the manuscript scientifically sound in its present form?
Yes

Are the interpretations and conclusions justified by the results?
Yes

Is the language acceptable?
Yes

Do you have any ethical concerns with this paper?
No

Have you any concerns about statistical analyses in this paper?
No

Reports © 2020 The Reviewers; Decision Letters © 2020 The Reviewers and Editors; Responses © 2020 The Reviewers, Editors and Authors. Published by the Royal Society under the terms of the Creative Commons Attribution License http://creativecommons.org/licenses/by/4.0/, which permits unrestricted use, provided the original author and source are credited

Recommendation?
Accept with minor revision (please list in comments)

Comments to the Author(s)
This manuscript is very well written, and discusses the critical and timely subject of pathogen spillover. Tehel and colleagues investigate the effects of possible viral spillover from honey bees to bumble bees by infecting bumble bees with three honey bee-derived viruses : black queen cell virus, and two variants of deformed wing virus. Methods include two different infection routes, injection and oral, and measured both viral loads and mortality of infected individuals, compared back to honey bees. Results indicate that bumble bees are indeed competent hosts (supported by previous research of field-collected bumble bees and laboratory assays), although this study did not observe any virulence effects from this infection. The authors do a good job of qualifying their virulence results in the discussion and appropriately incorporating all findings into the broader, complex field of pollinator virus ecology. This is a strong study which is important for the fields of bee health and pathogen spillover.

Minor discussion points :

Bumble bees and honey bees were injected with the same viral quantity, but honey bee workers tend to be smaller than bumble bee workers, and possibly experienced higher mortality due to a proportionally higher load. This may not be the case, though, as the resulting titers in honey bees were higher than bumble bees. It would be interesting to compare mortality differences between honey bees and bumble bees with equal resulting titers (if it is even possible to reach such high titers in bumble bees). The authors may want to add additional information in the discussion to address these points.

The authors properly address the importance of field-relevant conditions when assessing virus spillover. Total starvation is a very (possibly too) stressful situation, which may be why no differences were found (all bees were instead just perishing from starvation). Perhaps less extreme conditions (like poor nutrition versus absence of any nutrients whatsoever) could tease apart the virulence effects of the viruses. Furthermore, mortality is a fairly "stringent" response variable, and there could be sublethal effects which are not captured in the study. The authors may want to add additional information in the discussion to address these points.

Review form: Reviewer 2

Is the manuscript scientifically sound in its present form?
Yes

Are the interpretations and conclusions justified by the results?
Yes

Is the language acceptable?
Yes

Do you have any ethical concerns with this paper?
No

Have you any concerns about statistical analyses in this paper?
Yes

Recommendation?
Accept with minor revision (please list in comments)

Comments to the Author(s)
Experimental viral spill-over from honey bees to bumble bees: no immediate fitness costs but potential future threats to novel wild bee hosts.
Authors: Anja Tehel, Tabea Streicher, Simon Tragust, and Robert Paxton.
RSOS-200480

The article provides evidence of an effect of viral infection on the probability of survival in bumble bees infected with DWV-A, DWV-B and BQCV under laboratory conditions. It also analyzed the impact of a stressor (food depravation) on survival in infected bees. However, the study doesn't prove that there is a transmission of viruses from honey bees to bumble bees, but it does show that the viruses (DWV-A, DWV-B and BQCV) can replicate in bumble bees inoculated via injection and orally. It also shows that the viral genome copies in honey bees were higher compared to bumble bees.

The lines referenced are the single spaced line numbers.

Line 26, p. 2. I would suggest not using the term 'pandemic'. Klee et al. (2007) reported the presence of Nosema ceranae in samples form Europe, Brazil, New Zeeland, US and Vietnam. Although there are a number of reports from other locations, the OIE has not reported a worldwide spread of Nosema ceranae, there are a number of countries that have not tested or proven the presence of Nosema ceranae. Also, the correct term for a disease in an animal population is 'panzootic'.

Line 35, p.2. I would suggest caution when implying that honey bees are the source of pathogens, the references cited reported an association of diseases between honey bees and wild pollinators, but there is no proof that honey bees are the only source, it might be that wild pollinators act as reservoirs of diseases that affect honey bees.

Lines 37 and 41, p.2. I think you are referring to individual viruses and not the taxonomic entity, I would revise the nomenclature of the name of the viruses and the use of italics.

Line 42, p.2. Please revise the use of the world 'pandemic' through out the manuscript.

Lines 45-48, p.2. Arbetman et al. (2017) found that the bumble bees that were more susceptible to population decline were the ones not parasitized with C. bombi, Nosema spp, or L. bucneri. Also, Cameron and Sadd (2019) suggested a number of causes that could be involved in bumble bee population decline, not only parasites, but other pathogens (e.g. viruses) and abiotic stressors. I would suggest revising the references for the statement. Also, do the authors want to say 'parasites' or 'pathogens' for their argument?

Line 57 p.2. Fürst et al. (2014) recognized that their study could not prove the direction of the transmission, I would suggest caution when stating that the transmission of DWV occurs from honey bees to bumble bees.

Line 54 p.2. The primers used to amplify the target sequences is not clear. The authors mentioned that the qPCR was done as per Tehel et al (2019), were the primers the ones used by McMahon et al. (2015)?

Lines 14-15 p.4. It would be helpful to include the sequences of DWV-A and DWV-B.

Lines 33-44 p.6. I can see that the results of the Cox Proportional Hazard Model are in the Supplementary Table 2. However, it wouldn't hurt to put the p values in the results of '3.2.1 Bumble bees-fed inoculum, satiated' (just like they were written for 3.1).

Line 36 p. 6. Is there a rationale for using only abdomens for RNA extraction? DWV has tropism for different tissues, including the brain of bees.

Line 13 p. 7. What statistical method was used to analyze the differences in bee size between treatments?

Lines 34-35 p.8. I agree with the authors, based on the tests used in their study, caution must be taken to assume virulence (e.g. there were no tests of LD50 for each virus or tests of different concentration of inoculum on bee survival).

Line 57-60 p. 8. It's not clear to me what is the association between the capability of a virus to infect a new host, and the loss of 'viral fitness or virulence in the reservoir host'?

Observation on the discussion. Studies to assess the impact of pathogens -known to affect honey bees-, on wild pollinators are needed. There is a lack of information on pathogenicity, virulence and immune responses of wild pollinators to viral infections (and other pathogens). Also, most authors assume honey bees are transmitting the diseases to wild pollinators, but the same viruses have been diagnosed in wild pollinators (e.g. stingless bees), thus, more studies on the direction of the transmission are needed. Like mentioned above, I suggest to the authors to be careful on drawing conclusions on the direction of the viral transmission between honey bees and wild pollinators, and to reconsider the use of the term 'spill-over' in the title, and think of other terms such as 'experimental infection'.

I hope the authors find my suggestions helpful for their manuscript.

Decision letter (RSOS-200480.R0)

We hope you are keeping well at this difficult and unusual time. We continue to value your support of the journal in these challenging circumstances. If Royal Society Open Science can assist you at all, please don't hesitate to let us know at the email address below.

Dear Ms Tehel

On behalf of the Editors, I am pleased to inform you that your Manuscript RSOS-200480 entitled "Experimental viral spill-over from honey bees to bumble bees: no immediate fitness costs but potential future threats to novel wild bee hosts" has been accepted for publication in Royal Society Open Science subject to minor revision in accordance with the referee suggestions. Please find the referees' comments at the end of this email.

The reviewers and handling editors have recommended publication, but also suggest some minor revisions to your manuscript. Therefore, I invite you to respond to the comments and revise your manuscript.

• Ethics statement
If your study uses humans or animals please include details of the ethical approval received, including the name of the committee that granted approval. For human studies please also detail whether informed consent was obtained. For field studies on animals please include details of all permissions, licences and/or approvals granted to carry out the fieldwork.

• Data accessibility
It is a condition of publication that all supporting data are made available either as supplementary information or preferably in a suitable permanent repository. The data accessibility section should state where the article's supporting data can be accessed. This section

should also include details, where possible of where to access other relevant research materials such as statistical tools, protocols, software etc can be accessed. If the data has been deposited in an external repository this section should list the database, accession number and link to the DOI for all data from the article that has been made publicly available. Data sets that have been deposited in an external repository and have a DOI should also be appropriately cited in the manuscript and included in the reference list.

If you wish to submit your supporting data or code to Dryad (http://datadryad.org/), or modify your current submission to dryad, please use the following link:
http://datadryad.org/submit?journalID=RSOS&manu=RSOS-200480

• Competing interests
Please declare any financial or non-financial competing interests, or state that you have no competing interests.

• Authors' contributions
All submissions, other than those with a single author, must include an Authors' Contributions section which individually lists the specific contribution of each author. The list of Authors should meet all of the following criteria; 1) substantial contributions to conception and design, or acquisition of data, or analysis and interpretation of data; 2) drafting the article or revising it critically for important intellectual content; and 3) final approval of the version to be published.

All contributors who do not meet all of these criteria should be included in the acknowledgements.

We suggest the following format:
AB carried out the molecular lab work, participated in data analysis, carried out sequence alignments, participated in the design of the study and drafted the manuscript; CD carried out the statistical analyses; EF collected field data; GH conceived of the study, designed the study, coordinated the study and helped draft the manuscript. All authors gave final approval for publication.

• Acknowledgements
Please acknowledge anyone who contributed to the study but did not meet the authorship criteria.

• Funding statement
Please list the source of funding for each author.

Please ensure you have prepared your revision in accordance with the guidance at https://royalsociety.org/journals/authors/author-guidelines/ -- please note that we cannot publish your manuscript without the end statements. We have included a screenshot example of the end statements for reference. If you feel that a given heading is not relevant to your paper, please nevertheless include the heading and explicitly state that it is not relevant to your work.

Because the schedule for publication is very tight, it is a condition of publication that you submit the revised version of your manuscript before 29-May-2020. Please note that the revision deadline will expire at 00.00am on this date. If you do not think you will be able to meet this date please let me know immediately.

To revise your manuscript, log into https://mc.manuscriptcentral.com/rsos and enter your Author Centre, where you will find your manuscript title listed under "Manuscripts with Decisions". Under "Actions," click on "Create a Revision." You will be unable to make your revisions on the originally submitted version of the manuscript. Instead, revise your manuscript and upload a new version through your Author Centre.

When submitting your revised manuscript, you will be able to respond to the comments made by the referees and upload a file "Response to Referees" in "Section 6 - File Upload". You can use this to document any changes you make to the original manuscript. In order to expedite the processing of the revised manuscript, please be as specific as possible in your response to the referees. We strongly recommend uploading two versions of your revised manuscript:

1) Identifying all the changes that have been made (for instance, in coloured highlight, in bold text, or tracked changes);
2) A 'clean' version of the new manuscript that incorporates the changes made, but does not highlight them.

When uploading your revised files please make sure that you have:

1) A text file of the manuscript (tex, txt, rtf, docx or doc), references, tables (including captions) and figure captions. Do not upload a PDF as your "Main Document";
2) A separate electronic file of each figure (EPS or print-quality PDF preferred (either format should be produced directly from original creation package), or original software format);
3) Included a 100 word media summary of your paper when requested at submission. Please ensure you have entered correct contact details (email, institution and telephone) in your user account;
4) Included the raw data to support the claims made in your paper. You can either include your data as electronic supplementary material or upload to a repository and include the relevant doi within your manuscript. Make sure it is clear in your data accessibility statement how the data can be accessed;
5) All supplementary materials accompanying an accepted article will be treated as in their final form. Note that the Royal Society will neither edit nor typeset supplementary material and it will be hosted as provided. Please ensure that the supplementary material includes the paper details where possible (authors, article title, journal name).

Supplementary files will be published alongside the paper on the journal website and posted on the online figshare repository (https://rs.figshare.com/). The heading and legend provided for each supplementary file during the submission process will be used to create the figshare page, so please ensure these are accurate and informative so that your files can be found in searches. Files on figshare will be made available approximately one week before the accompanying article so that the supplementary material can be attributed a unique DOI.

Please note that Royal Society Open Science charge article processing charges for all new submissions that are accepted for publication. Charges will also apply to papers transferred to Royal Society Open Science from other Royal Society Publishing journals, as well as papers submitted as part of our collaboration with the Royal Society of Chemistry (https://royalsocietypublishing.org/rsos/chemistry).

If your manuscript is newly submitted and subsequently accepted for publication, you will be asked to pay the article processing charge, unless you request a waiver and this is approved by Royal Society Publishing. You can find out more about the charges at https://royalsocietypublishing.org/rsos/charges. Should you have any queries, please contact openscience@royalsociety.org.

Once again, thank you for submitting your manuscript to Royal Society Open Science and I look forward to receiving your revision. If you have any questions at all, please do not hesitate to get in touch.

Kind regards,
Andrew Dunn
Royal Society Open Science Editorial Office
Royal Society Open Science

openscience@royalsociety.org

on behalf of Dr Krijn Paaijmans (Associate Editor)
openscience@royalsociety.org

Associate Editor Comments to Author (Dr Krijn Paaijmans):

Comments to the Author:
Please pay attention to the following comment of reviewer #2 with regards to the statictical analysis: "Line 13 p. 7. What statistical method was used to analyze the differences in bee size between treatments?"

Reviewer comments to Author:
Reviewer: 1

Comments to the Author(s)
This manuscript is very well written, and discusses the critical and timely subject of pathogen spillover. Tehel and colleagues investigate the effects of possible viral spillover from honey bees to bumble bees by infecting bumble bees with three honey bee-derived viruses : black queen cell virus, and two variants of deformed wing virus. Methods include two different infection routes, injection and oral, and measured both viral loads and mortality of infected individuals, compared back to honey bees. Results indicate that bumble bees are indeed competent hosts (supported by previous research of field-collected bumble bees and laboratory assays), although this study did not observe any virulence effects from this infection. The authors do a good job of qualifying their virulence results in the discussion and appropriately incorporating all findings into the broader, complex field of pollinator virus ecology. This is a strong study which is important for the fields of bee health and pathogen spillover.

Minor discussion points :

Bumble bees and honey bees were injected with the same viral quantity, but honey bee workers tend to be smaller than bumble bee workers, and possibly experienced higher mortality due to a proportionally higher load. This may not be the case, though, as the resulting titers in honey bees were higher than bumble bees. It would be interesting to compare mortality differences between honey bees and bumble bees with equal resulting titers (if it is even possible to reach such high titers in bumble bees). The authors may want to add additional information in the discussion to address these points.

The authors properly address the importance of field-relevant conditions when assessing virus spillover. Total starvation is a very (possibly too) stressful situation, which may be why no differences were found (all bees were instead just perishing from starvation). Perhaps less extreme conditions (like poor nutrition versus absence of any nutrients whatsoever) could tease apart the virulence effects of the viruses. Furthermore, mortality is a fairly "stringent" response variable, and there could be sublethal effects which are not captured in the study. The authors may want to add additional information in the discussion to address these points.

Reviewer: 2

Comments to the Author(s)
Experimental viral spill-over from honey bees to bumble bees: no immediate fitness costs but potential future threats to novel wild bee hosts.
Authors: Anja Tehel, Tabea Streicher, Simon Tragust, and Robert Paxton.
RSOS-200480

The article provides evidence of an effect of viral infection on the probability of survival in bumble bees infected with DWV-A, DWV-B and BQCV under laboratory conditions. It also analyzed the impact of a stressor (food depravation) on survival in infected bees. However, the study doesn't prove that there is a transmission of viruses from honey bees to bumble bees, but it does show that the viruses (DWV-A, DWV-B and BQCV) can replicate in bumble bees inoculated via injection and orally. It also shows that the viral genome copies in honey bees were higher compared to bumble bees.

The lines referenced are the single spaced line numbers.

Line 26, p. 2. I would suggest not using the term 'pandemic'. Klee et al. (2007) reported the presence of Nosema ceranae in samples form Europe, Brazil, New Zeeland, US and Vietnam. Although there are a number of reports from other locations, the OIE has not reported a worldwide spread of Nosema ceranae, there are a number of countries that have not tested or proven the presence of Nosema ceranae. Also, the correct term for a disease in an animal population is 'panzootic'.

Line 35, p.2. I would suggest caution when implying that honey bees are the source of pathogens, the references cited reported an association of diseases between honey bees and wild pollinators, but there is no proof that honey bees are the only source, it might be that wild pollinators act as reservoirs of diseases that affect honey bees.

Lines 37 and 41, p.2. I think you are referring to individual viruses and not the taxonomic entity, I would revise the nomenclature of the name of the viruses and the use of italics.

Line 42, p.2. Please revise the use of the world 'pandemic' through out the manuscript.

Lines 45-48, p.2. Arbetman et al. (2017) found that the bumble bees that were more susceptible to population decline were the ones not parasitized with C. bombi, Nosema spp, or L. bucneri. Also, Cameron and Sadd (2019) suggested a number of causes that could be involved in bumble bee population decline, not only parasites, but other pathogens (e.g. viruses) and abiotic stressors. I would suggest revising the references for the statement. Also, do the authors want to say 'parasites' or 'pathogens' for their argument?

Line 57 p.2. Fürst et al. (2014) recognized that their study could not prove the direction of the transmission, I would suggest caution when stating that the transmission of DWV occurs from honey bees to bumble bees.

Line 54 p.2. The primers used to amplify the target sequences is not clear. The authors mentioned that the qPCR was done as per Tehel et al (2019), were the primers the ones used by McMahon et al. (2015)?

Lines 14-15 p.4. It would be helpful to include the sequences of DWV-A and DWV-B.

Lines 33-44 p.6. I can see that the results of the Cox Proportional Hazard Model are in the Supplementary Table 2. However, it wouldn't hurt to put the p values in the results of '3.2.1 Bumble bees-fed inoculum, satiated' (just like they were written for 3.1).

Line 36 p. 6. Is there a rationale for using only abdomens for RNA extraction? DWV has tropism for different tissues, including the brain of bees.

Line 13 p. 7. What statistical method was used to analyze the differences in bee size between treatments?

Lines 34-35 p.8. I agree with the authors, based on the tests used in their study, caution must be taken to assume virulence (e.g. there were no tests of LD50 for each virus or tests of different concentration of inoculum on bee survival).

Line 57-60 p. 8. It's not clear to me what is the association between the capability of a virus to infect a new host, and the loss of 'viral fitness or virulence in the reservoir host'?

Observation on the discussion. Studies to assess the impact of pathogens -known to affect honey bees-, on wild pollinators are needed. There is a lack of information on pathogenicity, virulence and immune responses of wild pollinators to viral infections (and other pathogens). Also, most authors assume honey bees are transmitting the diseases to wild pollinators, but the same viruses have been diagnosed in wild pollinators (e.g. stingless bees), thus, more studies on the direction of the transmission are needed. Like mentioned above, I suggest to the authors to be careful on drawing conclusions on the direction of the viral transmission between honey bees and wild pollinators, and to reconsider the use of the term 'spill-over' in the title, and think of other terms such as 'experimental infection'.

I hope the authors find my suggestions helpful for their manuscript.

Author's Response to Decision Letter for (RSOS-200480.R0)

See Appendix A.

Decision letter (RSOS-200480.R1)

We hope you are keeping well at this difficult and unusual time. We continue to value your support of the journal in these challenging circumstances. If Royal Society Open Science can assist you at all, please don't hesitate to let us know at the email address below.

Dear Ms Tehel,

It is a pleasure to accept your manuscript entitled "Experimental infection of bumble bees with honey bee associated viruses: no direct fitness costs but potential future threats to novel wild bee hosts" in its current form for publication in Royal Society Open Science.

Please ensure that you send to the editorial office an editable version of your accepted manuscript, and individual files for each figure and table included in your manuscript. You can send these in a zip folder if more convenient. Failure to provide these files may delay the processing of your proof. You may disregard this request if you have already provided these files to the editorial office.

You can expect to receive a proof of your article in the near future. Please contact the editorial office (openscience_proofs@royalsociety.org) and the production office (openscience@royalsociety.org) to let us know if you are likely to be away from e-mail contact -- if you are going to be away, please nominate a co-author (if available) to manage the proofing process, and ensure they are copied into your email to the journal.

Due to rapid publication and an extremely tight schedule, if comments are not received, your paper may experience a delay in publication. Royal Society Open Science operates under a continuous publication model. Your article will be published straight into the next open issue and

this will be the final version of the paper. As such, it can be cited immediately by other researchers. As the issue version of your paper will be the only version to be published I would advise you to check your proofs thoroughly as changes cannot be made once the paper is published.

Please see the Royal Society Publishing guidance on how you may share your accepted author manuscript at https://royalsociety.org/journals/ethics-policies/media-embargo/.

Thank you for your fine contribution. On behalf of the Editors of Royal Society Open Science, we look forward to your continued contributions to the Journal.

Kind regards,
Royal Society Open Science Editorial Office
Royal Society Open Science
openscience@royalsociety.org

on behalf of Dr Krijn Paaijmans (Associate Editor) and Andrew Dunn (Subject Editor)
openscience@royalsociety.org

Follow Royal Society Publishing on Twitter: @RSocPublishing
Follow Royal Society Publishing on Facebook:
https://www.facebook.com/RoyalSocietyPublishing.FanPage/
Read Royal Society Publishing's blog: https://blogs.royalsociety.org/publishing/

Appendix A

28.05.2020

Dear Editor,

We thank you and the anonymous referees for their positive and supportive reviews of our manuscript with the title: "Experimental viral spill-over from honey bees to bumble bees: no immediate fitness costs but potential future threats to novel wild bee hosts".

The reviewers have made very pertinent points, all of which we have taken on board and have addressed in our revision of the ms. The referees clearly have a good overview of the topic of the ms and, by incorporating revisions into the ms that address all of their points, we feel the ms is now more nuanced in discussing our data in relation to the views of others in the field. We thank the reviewers for the time they dedicated to their reviews, and for their perceptive and encouraging comments.

Below we detail our responses to the reviewers, point by point. We also upload a revised version of the ms, with changes tracked to facilitate re-reviewing of the ms. As you requested, we have introduced the additional ethics, competing interests and contributions sections to the end of the manuscript and have ensured that all data presented in the manuscript are openly available in the cited dryad repository.

All co-authors have seen the revised ms and agreed to it. We look forward to hearing back from you as to whether our changes are acceptable and await your decision of our revised ms for publication.

Yours sincerely,

Anja Tehel (on behalf of all co-authors)

Reviewer : 1

Comments to the Author(s)
This manuscript is very well written, and discusses the critical and timely subject of pathogen spillover. Tehel and colleagues investigate the effects of possible viral spillover from honey bees to bumble bees by infecting bumble bees with three honey bee-derived viruses: black queen cell virus, and two variants of deformed wing virus. Methods include two different infection routes, injection and oral, and measured both viral loads and mortality of infected individuals, compared back to honey bees. Results indicate that bumble bees are indeed competent hosts (supported by previous research of field-collected bumble bees and laboratory assays), although this study did not observe any virulence effects from this infection. The authors do a good job of qualifying their virulence results in the discussion and appropriately incorporating all findings into the broader, complex field of pollinator virus ecology. This is a strong study which is important for the fields of bee health and pathogen spillover.
****
Reply: We thank the reviewer for their positive and inspiring comments.
****

Minor discussion points :
Bumble bees and honey bees were injected with the same viral quantity, but honey bee workers tend to be smaller than bumble bee workers, and possibly experienced higher mortality due to a proportionally higher load. This may not be the case, though, as the resulting titres in honey bees were higher than bumble bees. It would be interesting to compare mortality differences between honey bees and bumble bees with equal resulting titres (if it is even possible to reach such high titres in bumble bees). The authors may want to add additional information in the discussion to address these points.
****
Reply: The size difference between honey bees and bumble bees is a valid issue raised by the referee though, as they themselves point out, the higher mortality of honey bees following viral infection is unlikely size-related because the resulting viral titres were higher in honey bees than bumble bees (under a purely size-titre relationship underpinning mortality across bee species, we would expect honey bees to die sooner *and* at lower titre than bumble bees). We have now added a sentence to the discussion to address the size difference between honey bees and bumble bees (lines 267-270).

We have undertaken several experiments in which we injected bumble bees but they always attained roughly similar viral titres after several days, which remained fairly constant (Supplementary Fig S2). We therefore cannot say if it would be possible for bumble bees to reach viral titres as high as in honey bees, though we suspect there is an upper asymptote (saturation) of viral titre per bee more or less independent of starting inoculum size. Maybe injecting more than 10^7 genome equivalents per bee would lead to higher infections but our former experiments with honey bee pupae have shown that when we injected 10^2 or 10^4 genome equivalents, no difference was detectable in viral titre after 3 days (Tehel et al. 2019), suggesting that hosts reach an upper titre (saturation). Therefore we suggest that, if the virus already establishes itself well and replicates, the starting concentration of the injection may not be that important in determining final viral titre. It might therefore be impossible to vary the resulting viral titre in a bee, whether honey bee or bumble bee. We have added an additional paragraph to the discussion to draw out these points, as recommended by the referee (lines 295-304).
****

The authors properly address the importance of field-relevant conditions when assessing virus spillover. Total starvation is a very (possibly too) stressful situation, which may be why no differences were found (all bees were instead just perishing from starvation). Perhaps less extreme conditions

(like poor nutrition versus absence of any nutrients whatsoever) could tease apart the virulence effects of the viruses. Furthermore, mortality is a fairly "stringent" response variable, and there could be sublethal effects which are not captured in the study. The authors may want to add additional information in the discussion to address these points.

****

Reply: Thank you for these additional points to consider in the discussion, which we have now done (lines 319-324).

For information, in our experiment under "benign" conditions we used as a food resource 50% sucrose solution instead of a diet based on carbohydrates as well as pollen, which would have been more natural and more nutritious. We found that feeding pollen resulted in workers becoming pseudo-queens and starting to lay eggs. Under these conditions, a comparison between workers that remained as workers and those that had become pseudo-queens might have been compromised due to possible changes in survival (e.g. up-regulation of innate immunity) due to reproduction. Therefore our 'benign' conditions already represented a poor nutrition scenario and we were nonetheless not able to see a difference in mortality in our main experiment.

****

Reviewer : 2

The article provides evidence of an effect of viral infection on the probability of survival in bumble bees infected with DWV-A, DWV-B and BQCV under laboratory conditions. It also analyzed the impact of a stressor (food depravation) on survival in infected bees. However, the study doesn't prove that there is a transmission of viruses from honey bees to bumble bees, but it does show that the viruses (DWV-A, DWV-B and BQCV) can replicate in bumble bees inoculated via injection and orally. It also shows that the viral genome copies in honey bees were higher compared to bumble bees.

****

Reply: We thank the reviewer for their detailed and perceptive comments. We also agree with them that we have not addressed transmission *per se*, and have adjusted the ms accordingly (details given below in response to specific points).

****

Line 26, p. 2. I would suggest not using the term 'pandemic'. Klee et al. (2007) reported the presence of *Nosema ceranae* in samples from Europe, Brazil, New Zeeland, US and Vietnam. Although there are a number of reports from other locations, the OIE has not reported a worldwide spread of *Nosema ceranae*, there are a number of countries that have not tested or proven the presence of *Nosema ceranae*. Also, the correct term for a disease in an animal population is 'panzootic'.

****

Reply: The term has been removed.

****

Line 35, p.2. I would suggest caution when implying that honey bees are the source of pathogens, the references cited reported an association of diseases between honey bees and wild pollinators, but there is no proof that honey bees are the only source, it might be that wild pollinators act as reservoirs of diseases that affect honey bees.

****

Reply: A lot of datasets point to the direction that honeybees seem to be the main source of virus. But the reviewer is correct that directionality of spill-over has not been proven because data are primarily correlational. We are now more cautious and have added additional caveats to paragraph 4

of the introduction and added a new sentence at its end: '*We note, though, that most data suggesting viral spillover from honey bees to bumble bees are correlational; directionality has rarely been demonstrated and wild bees may also be a source of infection for honey bees [24].'*
****

Lines 37 and 41, p.2. I think you are referring to individual viruses and not the taxonomic entity, I would revise the nomenclature of the name of the viruses and the use of italics.
****
Reply: Virus names are now not written any more in italic.
****

Line 42, p.2. Please revise the use of the world 'pandemic' throughout the manuscript.
****
Reply: Thank you for pointing out our error. We now replace the term pandemic with panzootic.
****

Lines 45-48, p.2. Arbetman et al. (2017) found that the bumble bees that were more susceptible to population decline were the ones not parasitized with *C. bombi, Nosema spp,* or *L. bucneri*. Also, Cameron and Sadd (2019) suggested a number of causes that could be involved in bumble bee population decline, not only parasites, but other pathogens (e.g. viruses) and abiotic stressors. I would suggest revising the references for the statement. Also, do the authors want to say 'parasites' or 'pathogens' for their argument?

****
Reply: We agreed with the reviewer that the cited references do not highlight the role of parasites in bumble bee decline and therefore (i) we replaced Arbetman et al 2017 with Meuss et al. 2011, which doies highlight the role of parasites, and (ii) we qualified our statement to '*with parasites being a potential cause of their decline*'.

In terms of definitions, 'parasite' is often used in the evolutionary ecology literature to refer to an organism that lives on or from another and causes harm to it. Moreover, the references we cite (Meeus et al. 2011, Cameron & Sadd 2019) refer to a broad range of parasites from the micro (viruses, bacterial) to Eukaryotic Protozoa and Metazoa. We therefore opt to stick with 'parasite'.
****

Line 57 p.2. Fürst et al. (2014) recognized that their study could not prove the direction of the transmission, I would suggest caution when stating that the transmission of DWV occurs from honey bees to bumble bees.
****
Reply: The referee is correct that Fürst et al. (2014) did not show directionality but inferred it from the correlation in prevalence of virus in honey bees and bumble bees plus the higher prevalence in the former. We have therefore added '*and higher prevalence in the former*', which was missing from our original ms. In relation to caution over directionality, we did phrase our sentence carefully with 'spill-over……has been inferred….'. Also, we added a sentence at the end of the paragraph, a caveat to the correlational nature of the data used to infer transmission from honey bees to bumble bees. We hope we have thereby been appropriately cautious in our wording over spill-over.
****

Line 54 p.2. The primers used to amplify the target sequences is not clear. The authors mentioned that the qPCR was done as per Tehel et al (2019), were the primers the ones used by McMahon et al. (2015)?

****

Reply: Yes, the same primers as in McMahon et al. 2015 where used. The information is now added to the Supplemental Methods section.

****

Lines 14-15 p.4. It would be helpful to include the sequences of DWV-A and DWV-B.

****

Reply: We (Tehel et al. 2019) give full details of the DWV consensus sequences, methods used to assemble them and links to the original Illumina (fasta-format) NGS source files. Tehel et al. is fully open access. It would therefore be superfluous for us to repeat the same details in this manuscript. To highlight their availability, we have therefore extended the sentence to: *'(see [47] for consensus sequences and the pipeline used to assemble them from NGS data as well as BioProject ID PRJNA515220 for the original NGS source files)'*.

****

Lines 33-44 p.6. I can see that the results of the Cox Proportional Hazard Model are in the Supplementary Table 2. However, it wouldn't hurt to put the p values in the results of '3.2.1 Bumble bees-fed inoculum, satiated' (just like they were written for 3.1).

****

Reply: Thanks for picking this up. We have now added the missing information (p-values) to the main manuscript.

****

Line 36 p. 6. Is there a rationale for using only abdomens for RNA extraction? DWV has tropism for different tissues, including the brain of bees.

****

Reply: You are absolutely right. The virus is not just in the abdomen but the abdomen shows very high viral titres in infected bees (Chen et al., 2006; Iqbal & Mueller, 2007; Yue & Genersch, 2005) and the digestive tract is sometimes even directly identified as one of the predominantly infected tissues (Fievet et al., 2006). When starting the experiment, we decided just to use the abdomen for viral quantification because (i) it would allow us to compare viral titres between injected *versus* fed bees (in the latter, we assume virus infection of the gut to be the norm) and (ii) to eliminate the possibility that problems would arise during RNA extraction due to contaminating pigments of host compound eyes. With our experiences working with bumble bees on this manuscript, we might in future use whole bees for viral detection. But for the current manuscript, and without the insight we have subsequently gained from it, it was important to stick to one method to be able to compare across the manuscript's datasets.

****

Line 13 p. 7. What statistical method was used to analyze the differences in bee size between treatments?

****

Reply: We used a linear model in R for testing if the intertegular distance (size of the bee) differed between treatments. This information is stated in the legend to Supplementary Figure S6 in the supplementary material.

****

Lines 34-35 p.8. I agree with the authors, based on the tests used in their study, caution must be taken to assume virulence (e.g. there were no tests of LD50 for each virus or tests of different concentration of inoculum on bee survival).
****
Reply: Thank you; we have been acutely aware of the need for caution in the interpretation of our results.

The referee is correct that we have not extensively tested for LD50 (or similar). Concentrations used in our studies were chosen on the basis of former studies. They are comparable to viral titres others have found, for example, in pollen samples and on flowers, suggesting they might be field-realistic.
****

Line 57-60 p. 8. It's not clear to me what is the association between the capability of a virus to infect a new host, and the loss of 'viral fitness or virulence in the reservoir host'?
****
Reply: In serial passage experiments in which a parasite adapts to a novel host species, a common outcome is that the parasite's virulence diminishes in the original host (reviewed in Ebert, 1998; Barrett et al. 2009; Elena, 2017). This attenuation of virulence can be so strong that the parasite can no longer infect the former host (reviewed in Ebert, 1998). To clarify this point, we now add to the end of the sentence: '*if viral adaptation to the novel host (Bombus) trades off with a loss of virulence in the original host (Apis)*' and we cite the above references in the following sentence.
****

Observation on the discussion. Studies to assess the impact of pathogens -known to affect honey bees-, on wild pollinators are needed. There is a lack of information on pathogenicity, virulence and immune responses of wild pollinators to viral infections (and other pathogens). Also, most authors assume honey bees are transmitting the diseases to wild pollinators, but the same viruses have been diagnosed in wild pollinators (e.g. stingless bees), thus, more studies on the direction of the transmission are needed. Like mentioned above, I suggest to the authors to be careful on drawing conclusions on the direction of the viral transmission between honey bees and wild pollinators, and to reconsider the use of the term 'spill-over' in the title, and think of other terms such as 'experimental infection'.
****
Reply: We agree with the referee that caution is needed, and we have tried to ensure we have been cautious and even-handed in our presentation of data. Given that we took virus from honey bees and experimentally infected bumble bees with it, we can be rather certain that we have looked at the consequences of potential spill-over. But the referee has correctly pointed out that the title of the manuscript is slightly misleading in that we did not experiment on transmission *per se*. We thank the referee for their perceptive comment. We therefore alter the title to:
'**Experimental infection of bumble bees with honey bee associated viruses: no direct fitness costs but potential future threats to novel wild bee hosts**'
****

I hope the authors find my suggestions helpful for their manuscript.
****
Reply: Yes, we have. Thank you for your perceptive comments and for impressing on us the need to show due caution in the interpretation of the literature and our own data.
****
